# Multi-Omics Analysis of *Vicia cracca* Responses to Chronic Radiation Exposure in the Chernobyl Exclusion Zone

**DOI:** 10.3390/plants12122318

**Published:** 2023-06-14

**Authors:** Viktoria Voronezhskaya, Polina Volkova, Sofia Bitarishvili, Ekaterina Shesterikova, Mikhail Podlutskii, Gilles Clement, Christian Meyer, Gustavo Turqueto Duarte, Maksim Kudin, Dmitrii Garbaruk, Larisa Turchin, Elizaveta Kazakova

**Affiliations:** 1 Russian Institute of Radiology and Agroecology, 249032 Obninsk, Russia; voronezhskaya.vs@gmail.com (V.V.); bitarishvili.s@gmail.com (S.B.); eshesterikova89@gmail.com (E.S.); mikhail.podlutskii@gmail.com (M.P.); 2 Independent Researcher, 2440 Geel, Belgium; 3 Institute Jean-Pierre Bourgin (IJPB), INRAE, AgroParisTech, Université Paris-Saclay, 78000 Versailles, France; gilles.clement@inrae.fr (G.C.); christian.meyer@inrae.fr (C.M.); 4 Belgian Nuclear Research Centre—SCK CEN, 2400 Mol, Belgium; gustavo.duarte@sckcen.be; 5 Polesye State Radiation—Ecological Reserve, 247618 Khoiniki, Belarus; max.kudin@mail.ru (M.K.); dima.garbaruk.77@mail.ru (D.G.); turchin2006@bk.ru (L.T.)

**Keywords:** *Fabaceae*, transcriptomics, proteomics, metabolomics, low doses, abiotic stress

## Abstract

Our understanding of the long-term consequences of chronic ionising radiation for living organisms remains scarce. Modern molecular biology techniques are helpful tools for researching pollutant effects on biota. To reveal the molecular phenotype of plants growing under chronic radiation exposure, we sampled *Vicia cracca* L. plants in the Chernobyl exclusion zone and areas with normal radiation backgrounds. We performed a detailed analysis of soil and gene expression patterns and conducted coordinated multi-omics analyses of plant samples, including transcriptomics, proteomics, and metabolomics. Plants growing under chronic radiation exposure showed complex and multidirectional biological effects, including significant alterations in the metabolism and gene expression patterns of irradiated plants. We revealed profound changes in carbon metabolism, nitrogen reallocation, and photosynthesis. These plants showed signs of DNA damage, redox imbalance, and stress responses. The upregulation of histones, chaperones, peroxidases, and secondary metabolism was noted.

## 1. Introduction

The radioactive contamination of the environment can have detrimental effects on living organisms, including plants. As sessile organisms, plants must cope with radioactive contamination through complex response pathways and metabolic adjustments. The effects of ionising radiation on plants can vary depending on the dose and duration of exposure and the characteristics of a plant species [1,2,3,4]. At low doses, radiation can stimulate plant growth and enhance photosynthesis, while at higher doses, it can lead to DNA damage, chromosomal aberrations, and mutations, ultimately affecting plant growth and reproduction [1]. Plants can undergo epigenetic changes in response to ionising radiation, leading to alterations in gene expression and phenotype [2,3]. The study of the biological responses of plants to chronic radiation exposure in radioactively contaminated areas is important for understanding the fundamental mechanisms of plant radiobiological responses and developing effective strategies to remediate radioactively contaminated sites.

The relevance of plant research in radioactively contaminated areas is twofold. First, plants can be used as bioindicators of radioactive contamination in the environment [5]. The morphological and physiological changes in plants exposed to ionising radiation can be used to monitor the extent of contamination and assess the effectiveness of remediation strategies. Second, plants can be used in phytoremediation, a process in which plants remove, stabilise, or detoxify radioactive elements from the soil or water [6,7,8]. Phytoremediation has several advantages over traditional remediation methods, such as being cost-effective, sustainable, and environmentally friendly [9]. However, the success of phytoremediation depends on several factors, including the plant species used, the level and type of contamination, and the local environmental conditions [9].

The genus *Vicia* belongs to the *Leguminosae* family, commonly known as vetches, and includes several plant species of agricultural importance. The genus comprises nitrogen-fixing plants with great potential for human and animal nutrition [10]. As green manure, *Vicia* can improve soil structure and plant nutrition [10] and mitigate climate change consequences as a substitute for synthetic fertilisers [11]. It harbours a large community of rhizospheric microorganisms, which can help to increase phytoremediation efficiency and make a remarkable difference in the ecological restoration of polluted soils due to *Vicia’s* dual role as a cover crop and phytoremediator plant [12]. In recent years, several studies have focused on the use of *Vicia* species for phytoremediation: *V. sativa* for phenols [13], Cd [14,15], and polychlorinated biphenyl [16]; *V. faba* for Cd [17,18], Pu [17,18], oil [19], and Sr [20]; *V. villosa* for As [21]; and *V. cracca* as a heavy metal accumulator for Cu, Fe, Zn, and Pb [22].

*V. cracca* is a hardy, fast-growing plant common in the Chernobyl exclusion zone (CEZ). In areas contaminated by uranium mill tailings and radium production wastes, these plants showed a significant increase in chromosome aberrations and a decreased survival rate of sprouts [23], outlining this species as a sensitive indicator of radiation exposure. Therefore, understanding the biological responses of *V. cracca* to ionising radiation is essential for its role as a bioindicator and for the possible use of the *Vicia* genus for phytoremediation purposes.

In recent years, multi-omics approaches have emerged as powerful tools for studying the responses of plants to environmental stresses, including pollution [24]. These approaches integrate data from different “omics” levels, such as genomics, transcriptomics, proteomics, and metabolomics, in order to comprehensively understand the biological processes and molecular mechanisms of plant responses to pollution. In the context of natural areas, multi-omics approaches can be used to study the responses of plants to a variety of pollutants [24,25,26]. This approach helps identify key molecular pathways and biomarkers that can be used to monitor and mitigate the effects of pollution on plants and the environment.

The current article presents an integrated multi-omics analysis of *V. cracca* plants inhabiting radioactively contaminated areas in the CEZ. This approach can provide a complete picture of the molecular changes in plants experiencing chronic low-dose radiation exposure and help identify key molecular pathways and potential biomarkers. Studies on *V. cracca* using the multi-omics approach can therefore contribute to a better understanding of the biological responses of plants to ionising radiation and the development of effective strategies for the phytoremediation of radioactively contaminated areas.

## 2. Results

### 2.1. Environmental Conditions in Experimental Plots

**Figure 1:**
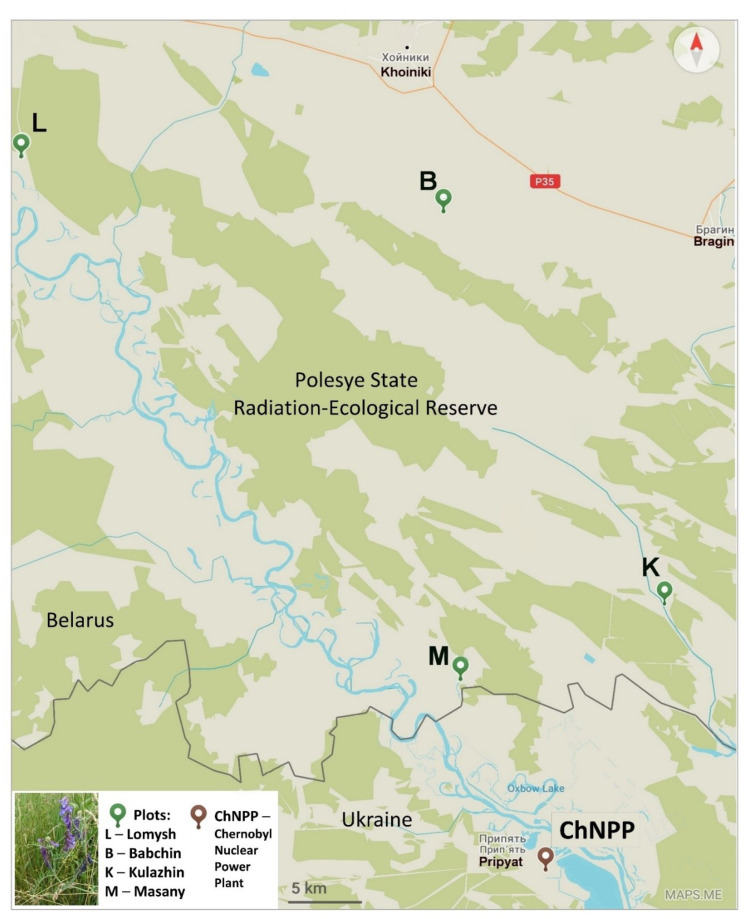
The location of experimental plots. ChNPP—Chernobyl NPP. **B**—reference plot, Babchin. **L**—reference plot, Lomysh. **K**—radioactively contaminated plot, Kulazhin. **M**—radioactively contaminated plot, Masany. Map was created using Maps.me service and modified in Microsoft PowerPoint 2021.

*Vicia cracca* leaves were sampled in the Polesye State Radiation-Ecological Reserve (Republic of Belarus) at four plots with different levels of radioactive contamination in June 2021. *V. cracca* leaves were sampled at Lomysh (**Lom**, **L**) and Babchin (**Bab**, **B**) as reference plots outside the CEZ and at two radioactively contaminated plots, Kulazhin (**Kul**, **K**) and Masany (**Mas**, **M**) (Figure 1).

The experimental plots had significantly different levels of radionuclide contamination (Table 1 and Table S1). The contaminated plots of Kulazhin and Masany were characterised by high ambient dose rates of 6.84 and 3.2 μSv × h^−1^, respectively. At the same time, the highest specific activity of ^137^Cs (36,990 Bq × kg^−1^) was observed in the Kulazhin plot. Specific activities of ^90^Sr were similar in both contaminated plots, being approximately 1500 Bq × kg^−1^ (Table 1 and Table S1). Additionally, we received data on the annual absorbed dose rates from thermoluminescent dosimeters in three plots out of four. The annual absorbed dose rates were calculated as mGy × year^−1^; reflected external γ-radiation exposure; and comprised 1.13 mGy for Babchin, 51.57 mGy for Kulazhin, and 27.51 mGy for Masany for 365 days.

The soil properties in the experimental plots were slightly different (Table 2): the soil samples in all plots were slightly acidic, with low humus content (1–5%) and low variation in the content of important minerals, being typical for soils of this region. Both control plots had lower humus content and lower related indices, such as hydrolytic acidity and available phosphorus and calcium compared to radioactively contaminated plots. Therefore, any stress-related findings shared for plots **M** and **K** cannot be directly attributed to lower soil nutrition compared to the controls, as the control soils were slightly poorer. The content of heavy metals in the soils in all the experimental plots did not exceed the permitted levels (Table S1).

### 2.2. Analysis of Expression of Selected Genes in V. cracca Populations

**Figure 2:**
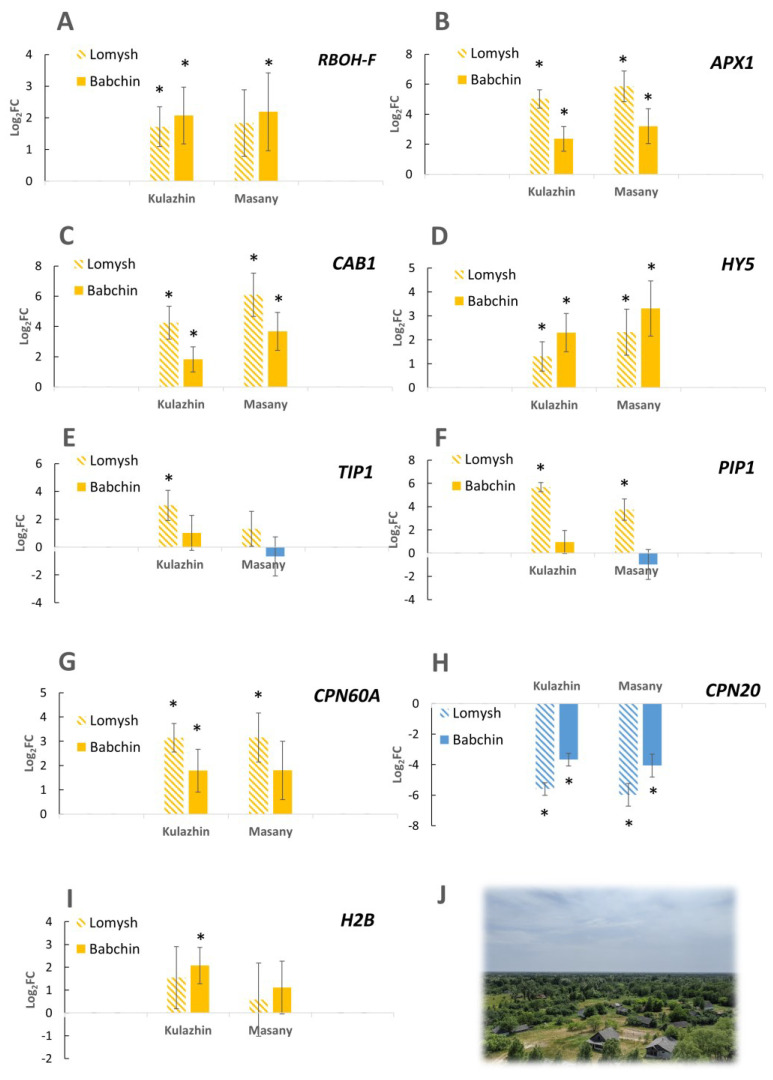
Log_2_FC for expression of selected genes in chronically irradiated *V. cracca* populations (Kulazhin and Masany) compared to each control (Lomysh and Babchin). (**A**)—NADPH-oxidase RBOH; (**B**)—ascorbate peroxidase APX1; (**C**)—chlorophyll-binding protein CAB1; (**D**)—transcription factor HY5; (**E**)—aquaporine TIP1; (**F**)—aquaporine PIP1; (**G**)—chloroplast chaperonin CPN60A; (**H**)—chloroplast chaperonin CPN20; (**I**)—histone *H2B.* *—log_2_FC ≥ |1|; *p*-value ≤ 0.05, Mann–Whitney U-test. (**J**)—view of the Chernobyl exclusion zone from the fire tower.

As a first approach, we performed the targeted gene expression analysis of some genes of interest revealed in our previous studies on other plant species [25,26,27]. The products of these genes participate in redox processes, signal transduction, and photosynthesis. Several genes encoding chaperones and histones were included to test the earlier observations of the possible role of these molecules in responses to chronic radiation [25,26,27]. Interestingly, the studied genes were mostly upregulated in the two contaminated plots compared to both controls (Figure 2). This observation included NADPH-oxidase *RBOH-F* (Figure 2A), ascorbate peroxidase *APX1* (Figure 2B), chlorophyll-binding protein *CAB1* (Figure 2C), transcription factor *HY5* (Figure 2D), histone *H2B* (Figure 2I), and chloroplast chaperonin *CPN60A* (Figure 2G). However, another chloroplast chaperonin *CPN20* was downregulated at both contaminated plots (Figure 2G). The differential expression of *V. cracca* genes is shown in Figure 2. These results match earlier patterns in other plant species in the Chernobyl exclusion zone [25,26,27]. While these gene functions seem to characterise a chronic ionising radiation response profile, the specific role of each of these genes on the adaptive responses to chronic radiation remains to be investigated.

### 2.3. Whole-Transcriptome Analysis of V. cracca Populations

**Figure 3:**
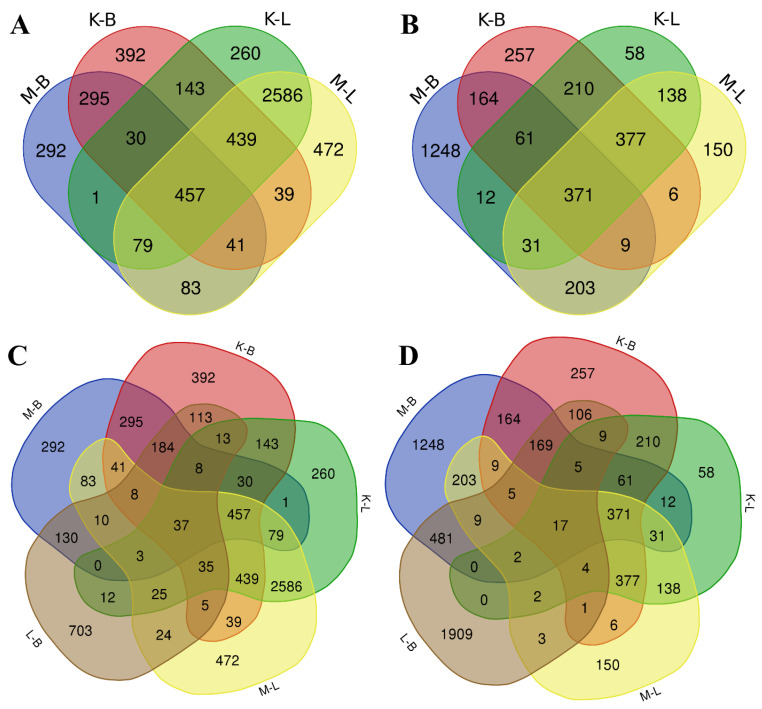
Venn diagrams for DEGs among all experimental plots. (**A**,**C**)—downregulated, (**B**,**D**)—upregulated. (**C**,**D**) include **L** × **B** comparisons between reference plots.

In order to estimate the whole mRNA transcriptional profile of *V. cracca* populations in conditions of chronic radiation exposure, we performed RNA sequencing of leaf samples. The *V. cracca* transcriptome was assembled de novo. After functional annotation, we revealed DEGs in samples from each radioactively contaminated plot compared to each control and performed the Gene Ontology analysis to functionally describe transcriptomic changes under chronic radiation exposure. The comparison of samples from **K** and **M** radioactively contaminated plots with each reference plot (**B** and **L**) revealed 4446 for **M** × **B** comparison, 4011 for **K** × **B**, 5426 for **K** × **L**, 5672 for **M** × **L**, and finally, 4033 for **L** × **B**. To exclude natural heterogeneity and possible confounding effects, we only used those genes which met the following conditions for further analysis: (1) had unidirectional differential expression compared to both controls and (2) were not differentially expressed between the two controls themselves. Among them were 457 downregulated DEGs and 371 upregulated DEGs (Table S2, Figure 3).

GO terms for DEGs shared for both radioactively contaminated plots compared to both controls are provided in Table 3. Upregulated DEGs were associated with nucleosomes and protein dimerisation, and downregulated DEGs were mainly photosynthesis-related.

For the in-depth analysis, we identified the 25 most downregulated and 25 most upregulated DEGs (Table 4). Downregulated DEGs imposed evident suppression of photosynthesis-related elements, specifically, the Calvin cycle, chlorophyll biogenesis, thiamine biosynthesis, and the translation process. The induction of peroxidases, protein catabolism machinery, phytohormonal relays, and cell-cycle-involved proteins was noted for upregulated genes. However, some elements of photosynthesis were found to be upregulated, which suggests an overall fine-tuning of the photosynthesis rate.

### 2.4. Non-Targeted Proteomic Analysis of V. cracca Populations

**Figure 4:**
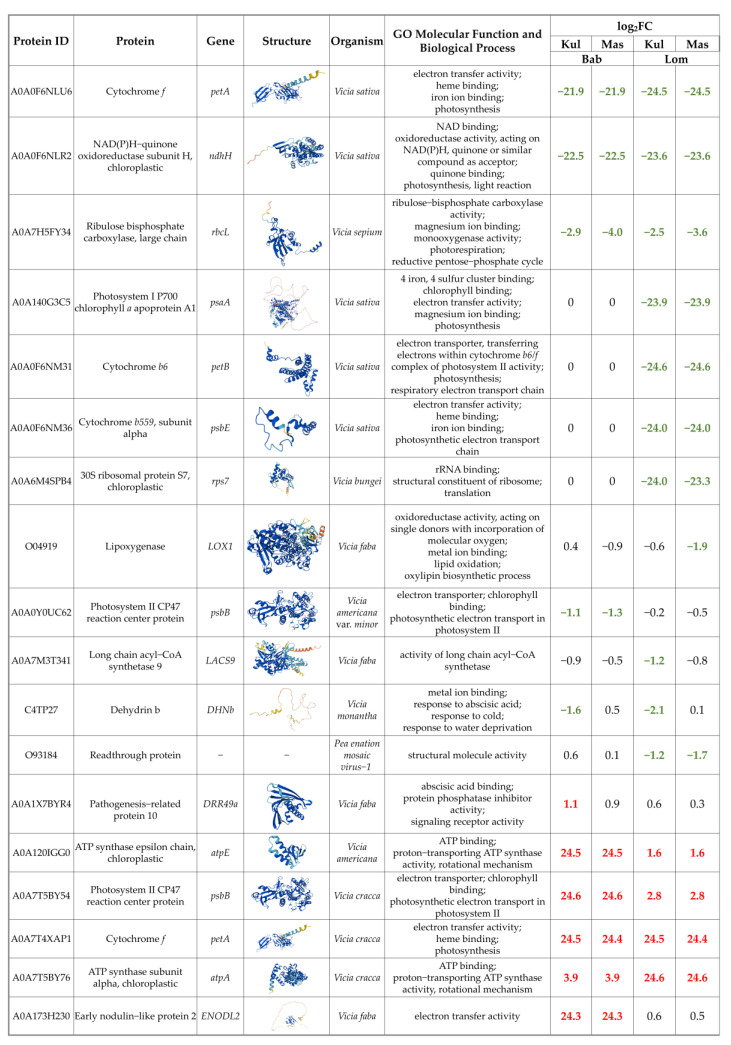
Proteins with differential abundance log_2_FC ≥ |1| for at least one radiation–control comparison.

We performed a non-targeted proteomic analysis to reveal changes in the protein profile of irradiated samples. One hundred and six proteins were successfully identified in the *V. cracca* samples (Table S3). Proteins whose abundance changed at least two-fold and unidirectionally in at least one of the “radiation × control” comparisons are highlighted in Figure 4. Interestingly, among them were proteins related to photosynthesis, ribosome assembly, and several cytochromes. Some proteins were only identified in the control samples (cytochrome *f* A0A0F6NLU, NAD(P)H-quinone oxidoreductase A0A0F6NLR2), while another cytochrome *f* A0A7T4XAP1 was only revealed in samples from radioactively contaminated plots.

**Figure 5:**
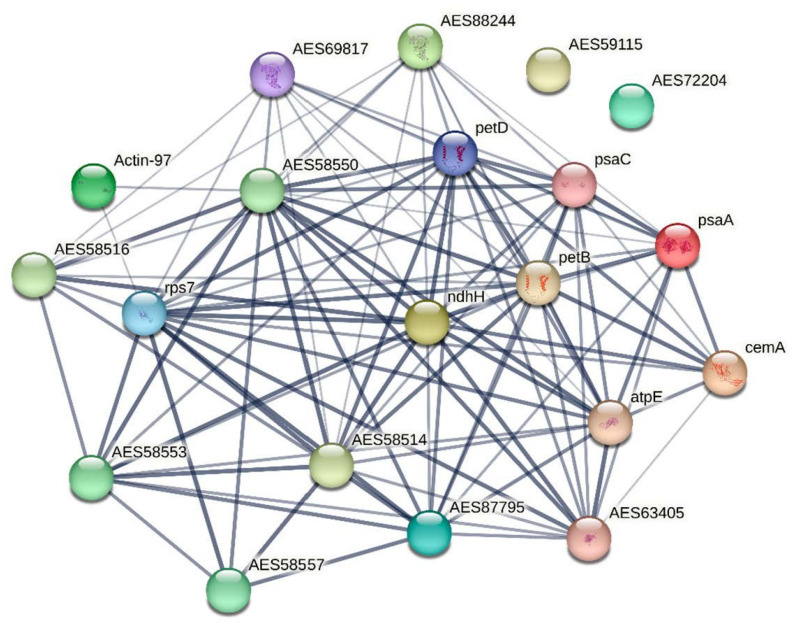
STRING summary network [29] for proteins with significantly different abundance between contaminated and reference plots (homologous proteins of *Medicago truncatula* L.). Line thickness indicates the strength of data support (confidence). psaA—photosystem I P700 chlorophyll *a* apoprotein; cemA—apocytochrome *f*; ndhH—NAD(P)-quinone oxidoreductase subunit I, chloroplastic; AES88244—ribulose bisphosphate carboxylase large chain domain protein; Actin-97—actin; AES72204—asparagine synthetase; AES87795—large subunit ribosomal protein L16; rps7—ribosomal protein S7; petD—cytochrome *b*; AES69817—ribulose 1,5-bisphosphate carboxylase, large subunit; psaC—4Fe-4S ferredoxin; AES63405—F-type H^+^/Na^+^-transporting ATPase subunit alpha; atpE—F-type H^+^/Na^+^-transporting ATPase subunit beta; petB H^+^/Na^+^—photosystem II chlorophyll *a* apoprotein; AES59115—plastocyanin-like domain protein; AES58557—ribosomal protein S3; AES58553—NADH-ubiquinone oxidoreductase; AES58516—NADH-ubiquinone oxidoreductase chain; AES58550—ATP synthase, subunit alpha; AES58514—ribosomal protein S12C.

In order to describe a possible functional interconnection between the identified proteins, we performed an analysis with the STRING tool (Figure 5), using homologous proteins of *Medicago truncatula* as an input (described in Section 4.7). The hubs of translational responses reflect the interconnection of photosynthetic responses and ribosomal proteins, suggesting profound changes in energy metabolism and translation. These results overlap with the transcriptomic data, further supporting the notion that a strategy for coping with chronic ionising radiation exposure involves the modulation of the photosynthesis rate, possibly for adjusting toxic ROS levels which are produced during energetic processes involving electron transport chains (ETCs), but also due to water radiolysis [2,25,26,28].

### 2.5. Non-Targeted Metabolomic Analysis of V. cracca Populations

**Figure 6:**
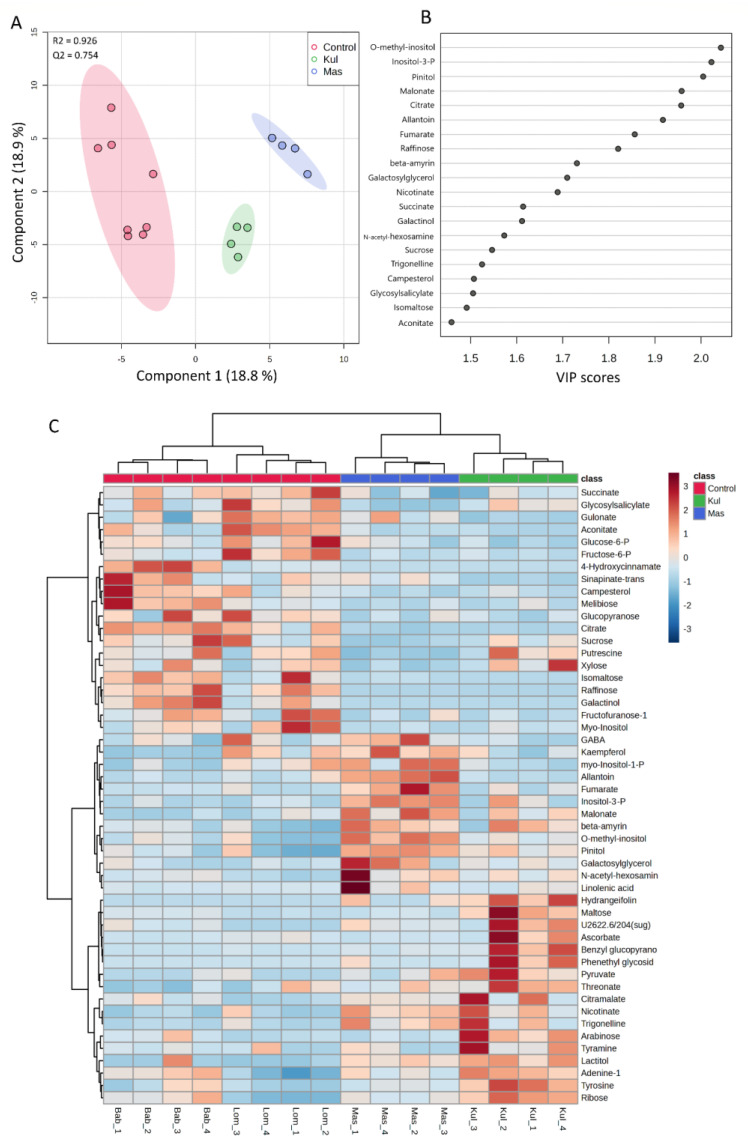
(**A**)—Score plots of partial least squares discrimination analysis (PLS-DA). (**B**)—A variable important in the projection (VIP) scores of the top 20 metabolites contributing to the separation of component 1 for metabolomic data of *V. cracca* from control and radioactively contaminated plots. (**C**)—Heatmap and the hierarchical cluster analysis for the top 50 detected metabolites in *V. cracca* samples from four study plots. Analysis was performed on normalised data, and Euclidean distances as the similarity measure and Ward’s linkage as the clustering algorithm were used. Glucose-6-P—glucose-6-phosphate; fructose-6-P—fructose-6-phosphate; GABA—*γ*-aminobutyric acid; *myo*-inositol-1-P—*myo*-inositol-1-phosphate; inositol-3-P—inositol-3-phosphate.

To specifically describe plant molecular phenotypes and to reveal the results of interactions of transcriptomes and proteomes, we performed a non-targeted GC-MS analysis of metabolites in *V. cracca* leaves. The obtained metabolic profiles included around 750 compounds, of which 70% were identified. We filtered the metabolites with close QC values for all replicates and selected 139 for further statistical analysis. Their concentrations are presented in Table S4. The normalised data were used for the PCA analysis. The scores plot (Figure S1) shows five principal components (PC1–PC5) explaining 74.2% of variations from the sixteen samples of *V. cracca*. According to PC1, which explains 24.1% of the variance, the metabolite profiles of samples from control plots were grouped separately from samples of contaminated plots, which overlapped. Supervised PLS-DA analysis (Figure 6) showed that samples from radioactively contaminated plots K and M were separated from control samples and each other by Component 1 (Figure 6A). The reliability of the PLS-DA model was confirmed using R^2^ (0.926) and Q^2^ (0.754) values obtained as a result of cross-validation. According to VIP scores, the greatest contribution to the separation was O-methyl-inositol, inositol-3-phosphate, and pinitol (Figure 6B).

To visualise the metabolomic changes in the control and chronically irradiated *V. cracca* plants, the top 50 metabolites were analysed using Heatmap and hierarchical cluster analysis. Obvious separation was observed between the control and chronically irradiated plants, where the four subclasses corresponded to the four studied plots (Figure 6C).

One-way ANOVA with Fisher’s least significant difference at the 5% confidence level was used to identify metabolites with different concentrations on radioactively contaminated plots. Data from two control plots, **L** and **B,** were combined and used as one group. As a result, 20 metabolites were identified in *V. cracca*, which were affected by chronic radiation exposure (Table 5).

**Figure 7:**
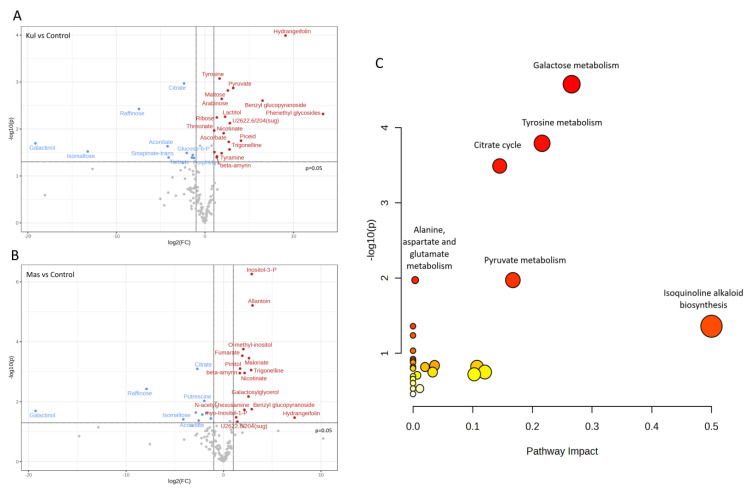
(**A**,**B**)—Volcano plot of the differentially abundant metabolites at Kulazhin (**A**) and Masany (**B**) compared to the control (**L + B**) with cut-offs for fold change |FC| > 2 and a *p*-value of < 0.05. Blue—downregulated metabolites, grey—non-significant, red—upregulated metabolites. (**C**) Plots depict several metabolic pathway alterations induced by chronic radiation exposure on experimental plots. The x-axis represents the pathway impact value from pathway topological analysis, and the y-axis is the −log of the *p*-value obtained from pathway enrichment analysis. Larger—log(p) and impact values characterised the most significantly changed pathways.

A total of 139 metabolites were used for volcano plot analysis to understand how metabolites change in each experimental plot relative to the control (Figure 7). Specifically, **K** had 10 significantly downregulated metabolites and 19 upregulated compared to control **L** + **B** (Figure 7A), while **M** had 11 downregulated metabolites and 15 upregulated (Figure 7B) with biological and statistical cut-offs for a fold change |FC| > 2 and a *p*-value of <0.05. Metabolites related to the citrate cycle, galactose metabolism, and glyoxylate and dicarboxylate metabolisms such as galactinol, raffinose, isomaltose, aconitate, and citrate were downregulated at both contaminated plots **K** and **M** compared to the control.

Kyoto Encyclopedia of Genes and Genomes (KEGG) identification numbers were used for pathway analyses of significantly changed metabolites (Table 5) in MetaboAnalyst 5.0 against the *Arabidopsis thaliana* library as a reference closest dicotyledonous plant in the database. Metabolic pathways altered considerably via chronic radiation exposure were revealed (Figure 7C), which included galactose metabolism (impact value: 0.27, FDR = 0.002), tyrosine metabolism (impact value: 0.22, FDR = 0.007), and citrate cycle (TCA cycle) (impact value: 0.15, FDR = 0.01) (Figure 7C).

### 2.6. Multi-Omics Data Integration

**Figure 8:**
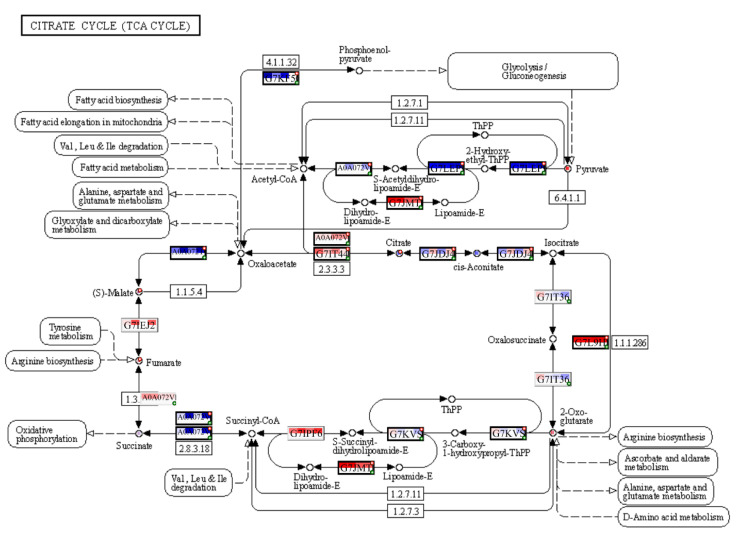
Enrichment for TCA cycle pathway in input datasets. A solid line presents a chemical reaction controlled by one or more enzyme. A dot line indicates adjacent metabolic pathway. Each Uniprot entry is divided by four squares, each representing a log_2_FC for a comparison between contaminated and reference plots, from left to right: **K** × **B**, **K** × **L**, **M** × **B**, **M** × **L**. The top line of the Uniprot entry is transcriptomic input, and bottom part is proteomic. The blue colour of a square reflects downregulation and red—upregulation. If a biological feature is significant for any of the omics data layers, it is highlighted by a thicker border and a red mark at the top right corner. A single databox can represent one or more biological features, where only the entry of the most significant feature will be shown. In this case, a green mark in the bottom right corner indicates one or more hidden features that share functions or contribute equally to the biological process. The metabolomic data can be seen as red or blue spots in the white circles, where usually, red means upregulation, and blue—downregulation. For more detailed data, please refer to Table S4. The figure was created with PaintOmics 4.

**Figure 9:**
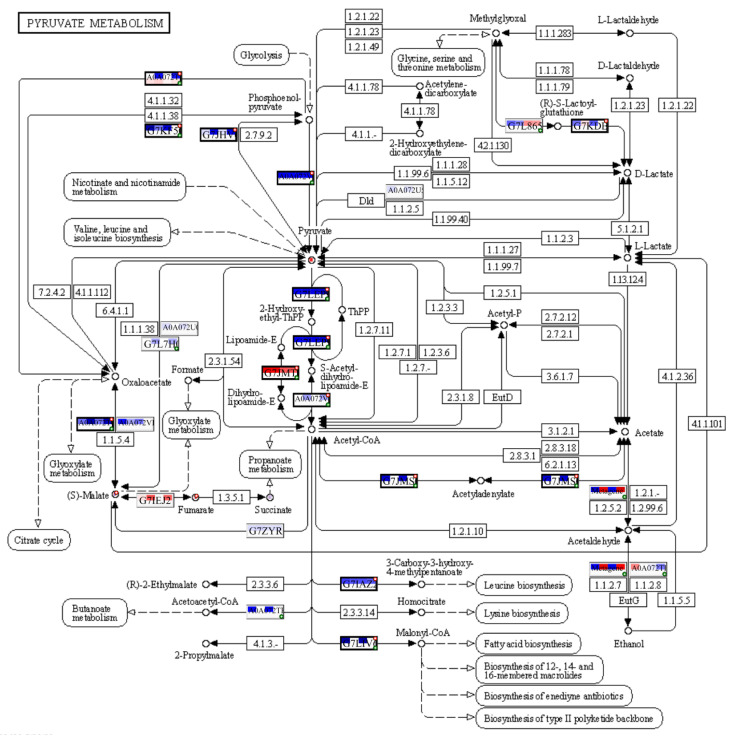
Enrichment for pyruvate metabolism pathway in input datasets. A solid line presents a chemical reaction controlled by one or more enzyme. A dot line indicates adjacent metabolic pathway. Each Uniprot entry is divided by four squares, each representing a log_2_FC for a comparison between contaminated and reference plots, from left to right: **K** × **B**, **K** × **L**, **M** × **B**, **M** × **L**. The top line of the Uniprot entry is transcriptomic input, and bottom part is proteomic. The blue colour of a square reflects downregulation, and red—upregulation. If a biological feature is significant for any of the omics data layers, it is highlighted by a thicker border and a red mark at the top right corner. A single databox can represent one or more biological features, where only the entry of the most significant feature will be shown. In this case, a green mark in the bottom right corner indicates one or more hidden features that share functions or contribute equally to the biological process. The metabolomic data can be seen as red or blue spots in the white circles, where usually, red means upregulation, and blue—downregulation. For more detailed data, please refer to Table S4. The figure was created with PaintOmics 4.

**Figure 10:**
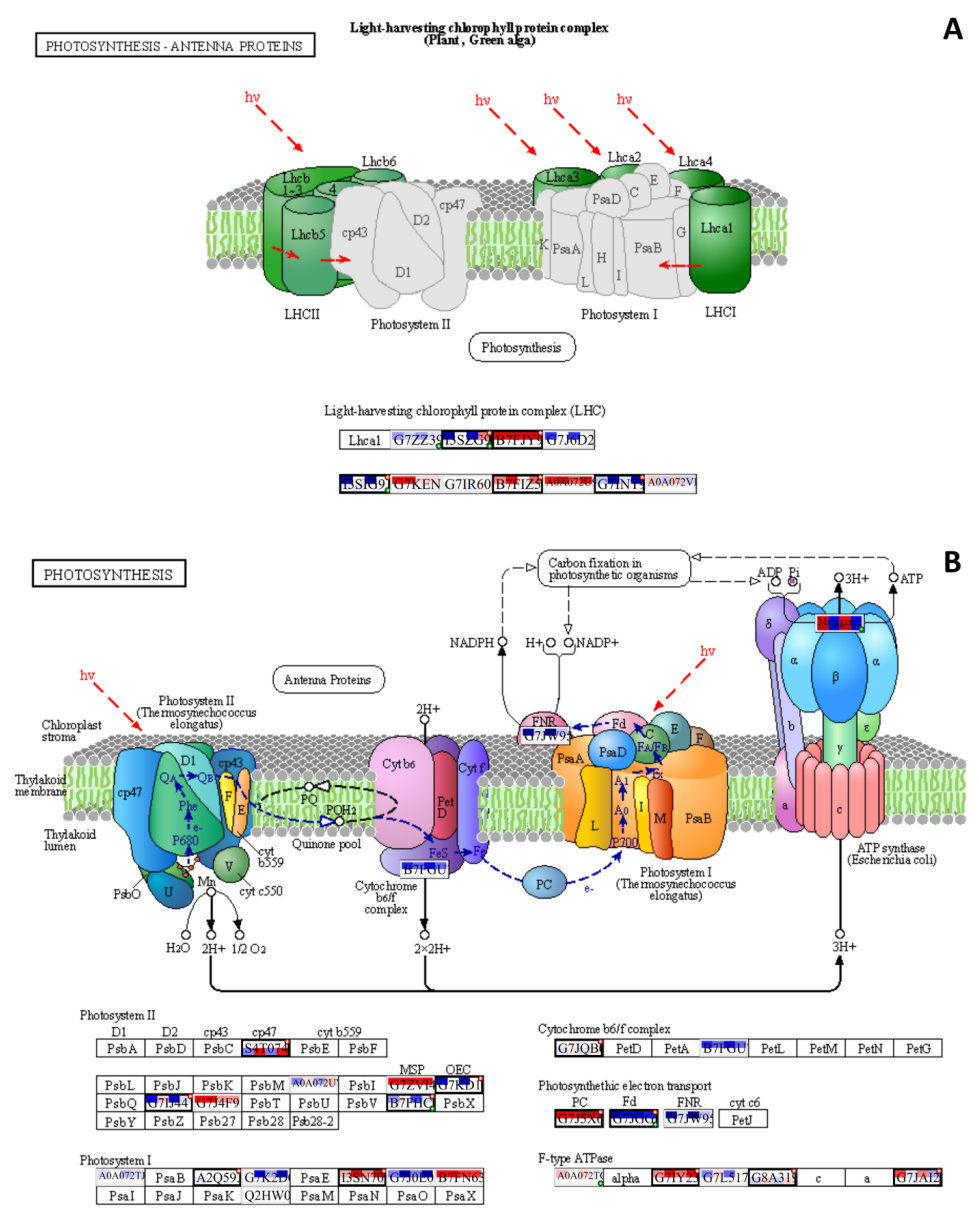
Enrichment for photosynthetic processes in input datasets ((**A**)—antenna proteins; (**B**)—electron transport chain). A solid line presents a chemical reaction controlled by one or more enzyme. A dot line indicates adjacent metabolic pathway. Each Uniprot entry is divided by four squares, each representing a log_2_FC for a comparison between contaminated and reference plots, from left to right: **K** × **B**, **K** × **L**, **M** × **B**, **M** × **L**. The top line of the Uniprot entry is transcriptomic input, and bottom part is proteomic. The blue colour of a square reflects downregulation, and red—upregulation. If a biological feature is significant for any of the omics data layers, it is highlighted by a thicker border and a red mark at the top right corner. A single databox can represent one or more biological features, where only the entry of the most significant feature will be shown. In this case, a green mark in the bottom right corner indicates one or more hidden features that share functions or contribute equally to the biological process. The metabolomic data can be seen as red or blue spots in the white circles, where usually red means upregulation, and blue—downregulation. For more detailed data, please refer to Table S4. The figure was created with PaintOmics 4.

The PaintOmics4 tool was used to identify pathways enriched or under-represented in contaminated plots on different functional levels for a holistic data view. Among them were general pathways of carbon (Figure S2) and fatty acid catabolism (Figure S3), as well as N-glycan biosynthesis (Figure S4). The multi-omics analysis confirmed changes in the citrate cycle, where fumarate, 2-oxoglutarate, and malonate accumulation was observed and citrate concentrations decreased (Figure 8), and in pyruvate metabolism (Figure 9), where the accumulation of pyruvate was accompanied by the downregulation of glycolysis in general (Figure 9 and Figure S2). Profound changes in photosynthesis were revealed (Figure 10). Among more specific pathways, we observed the upregulation of the glyoxylate cycle (Figure S5), the downregulation of biotin metabolism (Figure S6), and the upregulation of certain branches of propanoate metabolism, which led to β-alanine synthesis (Figure S7). Structural modifications of ribosomes were also evident (Figure S8), accompanied by significant changes in RNA polymerase subunits (Figure S9). Altogether, the results suggest that the modulation of energy production is a central aspect of plants growing under chronic radiation exposure.

## 3. Discussion

### 3.1. Carbon Metabolism and Photosynthesis

*V. cracca* plants growing in radioactively contaminated plots have differences in their carbon cycle metabolism compared to plants from control areas, including the upregulation of pyruvate (Figure 7 and Figure 9) and fumarate (Figure 7 and Figure 8) biosynthetic processes, which points to energetic and plastic rearrangements. This can be related to the decreased protein abundance of asparagine synthase (Figure 4), as asparagine-synthesis-deficient *A. thaliana* mutants showed an accumulation of alanine, GABA, pyruvate, and fumarate, indicating an alanine formation from pyruvate through the GABA shunt to consume excess ammonium in the absence of asparagine synthesis [30]. Indeed, alanine, aspartate, and glutamate metabolism are also enriched in our data (Figure 7). On the other hand, the decreased citrate concentrations (Figure 7) coupled with the upregulation of transcripts involved in its biosynthesis (Figure 8) points to several bypass possibilities of TCA due to redox changes in the cell. Organic acids represent the stored pools of fixed carbon. When the redox level in the cell increases, the TCA cycle in mitochondria is transformed into a partial cycle supplying citrate for the synthesis of 2-oxoglutarate and glutamate (citrate valve). At the same time, malate is accumulated and participates in the redox balance in different cell compartments (via the malate valve) [31,32]. Indeed, multi-omics analysis revealed higher 2-oxoglutarate concentrations and malate accumulation (Figure 8), which may support the activation of citrate and malate valves. The upregulation of the glyoxylate cycle intermediates, such as D-glycerate and glycolate (Figure S5), also suggests that the cells are trying to bypass some steps of the TCA cycle to generate key metabolic intermediates. This could represent a response to the decreased availability of other metabolic intermediates, such as citrate [31].

Fatty acids are major components of cell membranes, and changes in their biosynthesis could alter the fluidity and permeability of the membranes. The downregulation of pathways leading to fatty acid biosynthesis (Figure S3) suggests that the cells may be reducing their production of fatty acids in response to chronic radiation exposure. The downregulation of fatty acid biosynthesis pathways may have implications for the overall lipid metabolism and membrane composition of plant cells. The decrease in the production of propanoyl-CoA from 2-oxobutanoate (Figure S7) also confirms lipid metabolism downregulation in irradiated plants. The increase in the production of malonate semialdehyde from 3-hydroxy-propionyl-CoA (Figure S7) suggests that the cells may be relying more heavily on the degradation of odd-chain fatty acids or amino acids for energy production [33].

The reallocation of carbon can be seen as a profound change in galactose metabolism (Figure 7), indicated by the decrease in raffinose family oligosaccharide (RFO) concentrations. RFOs are involved in antioxidant, membrane-stabilising, and signalling pathways [34]. However, the reduction in galactinol and raffinose concentrations may rather be associated with their alleged role in providing carbon skeletons to lipid synthesis or thylakoid membrane biogenesis [35]. The further depletion of some carbohydrates was also confirmed by the downregulation of various N-glycans (Figure S4), which are involved in N-glycosylation and participate in multiple processes, including development, protein folding, photosynthesis, and phytohormone homeostasis [36]. N-glycans and raffinose family oligosaccharides interact with lectins in response to pathogens to initiate defence reactions [37]. It is plausible to suggest that the downregulation of raffinose and N-glycan metabolism may be partially attributed to the attempt to avoid an over-reaction to the continuous damage of cell wall structures by ionising radiation, which can be perceived as a pathogen attack and promote defence responses.

Changes in carbon availability can be related to decreased capacities of carbon assimilation. The chloroplasts of plants are particularly sensitive to ionising radiation due to the susceptibility of their light-absorbing pigments and genetic material to the negative effects of reactive oxygen species (ROS) [38,39,40]. Several photosynthesis-related genes were downregulated (Table 4). Changes in the antenna complex LHCII and photosystems I and II (Figure 10) were observed, which suggests that the photosynthetic machinery may undergo alterations in response to chronic radiation exposure. Downregulated DEGs included *ISPE_SOLLC*, *PUR5_VIGUN*, *PGL1B_ARATH*, *FTSI1_ARATH*, *CHLM_ARATH*, and *PGKH_WHEAT* (Table 4), which are associated with chloroplast development and chlorophyll biosynthesis [41,42,43,44,45]. Decreased expression can be either associated with redox damage of the ETC or developmental changes under chronic irradiation conditions. The reduced synthesis of cytochrome *b6*/*f* (Figure 4 and Figure 10B) and changes in ETC components also suggest a decrease in the efficiency of photosynthesis. The changes in Fd, FNP, and PC (Figure 10B) further confirm ETC dynamics alterations, which may impact the overall ATP production. The upregulation of ATPase components may indicate an increase in the cellular demand for ATP (Figure 10B), possibly due to the decreased efficiency of other energy-generating pathways such as glycolysis, which is evidently downregulated (Figure 9). Other signs of chloroplast protection and stabilisation were noted in the targeted gene expression analysis, where we observed the upregulation of the *CAB1* homologue (Figure 2), which is associated with chlorophyll accumulation and the stability of chloroplast membranes [46]. The expression of transcription factor *HY5*, which regulates, particularly, *CAB1* expression [47] and is a key factor of chlorophyll accumulation, was increased in all contaminated plots (Figure 2). The induction of these genes is aligned with our previous results [25,27] and points to the important role of HY5 in response to chronic radiation and the possible disruption of the circadian rhythms of irradiated plants [47,48,49].

Overall, these changes in energy metabolism suggest that the cells of plants growing in radioactively contaminated areas undergo significant alterations, possibly redirecting metabolic intermediates of the TCA cycle for the synthesis of protective compounds and relying more on fatty and amino acid catabolism for energy production than plants from the reference plots.

### 3.2. Chronic Radiation Stress Response: DNA Damage and Changes in the Redox Status

Chronic radiation exposure is a mutagenic factor capable of inducing DNA single- and double-strand breaks. Our previous research, however, revealed a very subtle DNA repair machinery response in plants growing under chronic radiation, such as *Pinus sylvestris* [25] and *Capsella bursa-pastoris* [26]. The deep sequencing of the *V. cracca* transcriptome revealed upregulated DEGs involved in DNA repair and cell cycle control (Table 4). Among most upregulated were topoisomerase 3-alpha *TOP3A_ARATH*, involved in homologous recombination, and cyclin *CCNB1_MEDSA*. Additionally, polymerase gamma 1 and damaged DNA-binding protein 1A (Table S2) were upregulated in radioactively contaminated plots. However, the canonical DNA damage response elements (such as ATR, ATM, and SOG1 [2]) were not identified, which may indicate the involvement of the identified DEGs in replication maintenance rather than radiation-caused strand break repair. Nevertheless, whether chronic radiation exposure affects DNA replication efficiency remains to be investigated. The upregulation of histones (Figure 2 and Table 4 and Table S2) and nucleosomal processes (Table 3) converge with our previous hypothesis of the role of DNA packaging in coping with chronic DNA damage rather than keeping energy-demanding systems of DNA damage recognition upregulated [25,26]. Although homologous recombination is a part of the response to DNA double-strand breaks [2], other components of DNA damage response signalling [50] were not modulated in our data (Table S2). However, a potential inducer of apoptosis, CEP1_ARATH [51], was strongly upregulated (Table 4), which would be expected in the case of increased DNA damage. Therefore, the data point to at least moderate DNA damage in plants from radioactively contaminated plots.

The chronic damage of macromolecules implies the need to protect DNA and protein integrity and conformation. Based on our previous findings of the involvement of histones and chaperones in response to chronic radiation exposure [25,26], we performed targeted expression analyses of several chaperones (Figure 2). The upregulation of the chloroplast chaperonin *CPN60A* homologue may be connected to decreased RuBisCO abundance (Figure 4). Rubisco deactivation may be a protective strategy under stress conditions, while CPN60A, which is involved in carboxylation, participates in photosynthesis maintenance under stress conditions, being upregulated, particularly under heat stress [52]. However, the *CPN20* homologue encoding another chloroplast chaperonin was significantly downregulated in both radioactively contaminated plots (Figure 2). CPN20 negatively regulates certain branches of ABA signalling [53,54]; therefore, its downregulation may be connected to *CPN20* signalling function rather than protein folding.

Finally, the redox imbalance in *V. cracca* is not only confirmed via TCA bypassing and photosynthesis reduction. The upregulation of peroxidases (Table 4 and Figure 2) suggests increased ROS concentrations. The downregulation of biotin metabolism (Figure S6) may lead to biotin deficiency, which, in turn, is associated with H_2_O_2_ accumulation [55]. In turn, biotin deficiency can contribute to observed changes in carbon metabolism since biotin is a cofactor for some carboxylases, decarboxylases, and transcarboxylases participating in fatty acid and carbohydrate metabolism [56]. Under radiation-induced ROS imbalance, accumulating secondary metabolites with antioxidant properties [1] is a promising coping strategy for chronic oxidative stress.

### 3.3. Secondary Metabolites

Indeed, several DEGs involved in the biosynthesis of phenolic compounds, which are important antioxidants, were induced in response to ionising radiation (Table S2), including *PALY_MEDSA* (Table 4), phenylalanine ammonia-lyase. This enzyme catalyses the first synthesis step of various phenylpropanoid compounds [57] and is often induced by ionising radiation [58,59,60]. The upregulation of the *HY5* transcription factor gene (Figure 2) can also induce anthocyanin biosynthesis [47]. Meanwhile, the downregulation of genes involved in terpenoid biosynthesis, such as *ISPE_SOLLC* and *CHLP_ARATH* (Table 4), potentially indicates a compensatory role in the biosynthesis of other metabolites. In *V. cracca* from contaminated plots, increased concentrations of metabolites involved in tyrosine metabolism were observed (Figure 7). Tyrosine is a precursor of metabolites that perform antioxidant functions (tocopherols and rosmarinic acid), electron transfer in the electron transport chain (plastoquinone and ubiquinone), and plant protection against pathogens (alkaloids and glycosides) [61].

Nitrogen reallocation is essential for nitrogen-containing secondary metabolites’ biosyntheses such as alkaloids, cyanogenic glycosides, glucosinolates, and nonprotein amino acids [62]. Besides general changes in amino acid metabolic pathways, we observed a significant increase in the allantoin concentration in the Masany contaminated plot (Table 5), with the highest ambient dose rate and activities of ^137^Cs and ^90^Sr in soil. Allantoin is an intermediate product of the purine catabolic pathway, which acts in nitrogen metabolism and transport in plants [63] and accumulates during environmental challenges, increasing stress tolerance [64]. In chronically irradiated *V. cracca* plants, an increase in the concentrations of nicotinate and its derivative trigonelline was revealed (Figure 7), which may have been associated with oxidative damage to cells and perturbations in cellular energy metabolism [65]. Several other secondary metabolites had a higher concentration in radioactively contaminated plots (Figure 7), including compounds with antioxidant and protective properties, such as pinitol [66], trigonelline [67], β-amyrin [68], hydrangeifolin [69], and piceid [70]. Therefore, at least partially, certain general metabolic pathways can be suppressed due to carbon and nitrogen reallocation for protective compound synthesis in plants growing in radioactively contaminated plots.

### 3.4. Protein Catabolism and RNA Processing

The changes in ribosomal protein synthesis (Figure S8) suggest a complex stress response in plants growing in radioactively contaminated plots, with multidirectional changes in the expression of ribosomal proteins. Such differential regulation patterns can be related to tissue-specific responses [71] or be an example of multidirectional ribosomal protein responses, as was shown for other stressors [72,73]. For instance, ribosomal proteins of large ribosomal subunits are highly responsive to stress and signalling molecules, indicating that they may have roles in stress mitigation besides a housekeeping function [72]. The extraribosomal functions of some ribosomal plant proteins include microRNA biogenesis, anti-virus defence, and plant immunity, which may be associated with the widespread duplication of ribosomal protein genes in plants [74].

The changes in RNA polymerase biosynthesis are also multidirectional (Figure S9). Eukaryotic RNA polymerase II components are mostly upregulated (Figure S9), while this enzyme complex is responsible for the transcription of protein-coding genes, long non-coding RNAs, and small RNAs [75]. The downregulation of RNA polymerase I and III components (Figure S9) may suggest a decrease in the transcription of rRNA and tRNA genes [75]. The processing of rRNA is modulated on the transcriptional level (Table 4). Overall, the changes in ribosomal protein synthesis and RNA polymerases suggest that the cells undergo significant alterations in their gene expression and translational patterns in response to chronic radiation exposure.

Plant growth and development are directly linked to ribosome synthesis and protein translation. Still, the precise molecular mechanisms and signals that connect cellular and environmental conditions with rRNA synthesis and ribosome assembly remain to be uncovered [76]. The ribosomal responses of *V. cracca* to chronic irradiation may be of particular interest since, lately, ribosomal proteins have been suggested as a valuable resource for manipulating the stress tolerance of crops [72].

## 4. Materials and Methods

### 4.1. Experimental Plots and Soil Sampling

Sampling was conducted at the Polesye State Radiation-Ecological Reserve (Republic of Belarus) in four plots with different levels of radioactive contamination in June 2021. *V. cracca* leaves were sampled in Lomysh (**Lom**, **L**) and Babchin (**Bab**, **B**) as reference plots outside the CEZ and in two radioactively contaminated plots, Kulazhin (**Kul**, **K**) and Masany (**Mas**, **M**) (Figure 1).

In each experimental plot, the ambient dose rate was determined using a dosimeter–radiometer MKS-02SA1 (SNIIP, Russia) at 1 m from the surface, and α- and β-particle flux densities (min^−1^ × cm^−2^) were determined 0.2 cm above the ground. Soil samples in each plot were collected using the envelope method from a rectangular area (1 × 2 m, a prick at each corner and a prick in centre) at a depth of up to 15 cm, and then, 5 pricks were mixed into a pooled sample (250–300 g). The activity concentrations of ^137^Cs in the soil samples were measured with a γ-spectrometer CANBERRA (Toledo, OH, USA). We used the radiochemical method to determine ^90^Sr-specific activity in soil sampling. To estimate the annual absorbed dose of external γ-radiation at each experimental plot, we installed a thermoluminescent dosimeter DTL-02 (NPP Doza, Moscow, Russia). The dosimeters were covered from weather exposure by a section of plastic tube installed over them and laid on the ground inside the perimeter of a sample plot.

At each plot, the soil properties and levels of heavy metal contamination were assessed, including pH; hydrolytic activity; cation exchange capacity; and K, P, Ca, Mg, Na, and humus content following the ISO standard for soil quality [77]. The total concentrations of heavy metals (Cd, Cu, Co, Ni, Cr, Mn, Pb, Zn, As, and Mo) in the soil samples were measured using a plasma optical emission spectrometer (ICP-OES, Australia) in accordance with the ISO 11047 standard following treatments with a mixture of HNO_3_, HCl, and HF [78].

### 4.2. Plant Sampling

*V. cracca* was sampled in the four plots by collecting pools of leaves from 5 to 7 plants (approximately 0.5 g in total). Sampled plants visually belonged to the same developmental stages. The samples were immediately frozen in liquid nitrogen until analyses were carried out. For quantitative real-time PCR (qRT-PCR) gene expression analysis, we used 4 samples per plot. For transcriptome, proteome, and metabolome analyses, *V. cracca* leaves were sampled from the same plants for all three analyses to ensure correlation among all omics levels. For the transcriptomic analysis, we used 3 samples per plot. For proteomic analysis, we used 4 samples from each contaminated plot and 3 from each reference plot. For metabolomic analysis, 4 samples from each reference and radioactively contaminated plot were used. Sampling was performed around noon for all experimental plots (Table S1). The samples were collected under similar environmental conditions, and no rain occurred during the sampling period.

### 4.3. Gene Expression by qRT-PCR

Based on our previous research using other plant species growing in the CEZ [25,26,27] and data from other authors on plant responses to chronic radiation exposure [79,80], we selected several genes to study specific transcriptional responses of *V. cracca* to chronic irradiation (Table 6). The products of these genes are associated with the antioxidant system, signal transduction, and photosynthesis processes. The expression of genes encoding aquaporins was also studied, as these proteins maintain the redox balance of cytosol and hydrogen peroxide transport [81]. To test the hypothesis of the essential role of chaperones and histones in the adaptive response to chronic irradiation, firstly raised in *Pinus sylvestris* L. [25] and *Capsella bursa-pastoris* L. [26], we included several genes encoding chaperones and histones for *V*. *cracca* in the analysis. *CYP2* and *ELF1A* [82] were used as reference genes.

For primer design, cDNA sequences of *A. thaliana* were aligned against *Fabaceae* sequences using the NCBI BLAST tool. For each target gene, 5–10 sequences of homologous genes with the highest score were selected. The sequences were processed in Clustal Omega 1.2.2, revealing the conserved regions. Next, primers to the selected regions were constructed using Primer BLAST. The specificity of primer pairs was tested using qRT-PCR and 2% agarose gel separation. Primer sequences are provided in Table S5.

Total RNA from *V. cracca* leaves was isolated using a GeneJET Plant RNA Purification Mini Kit (Thermo Fisher Scientific, Waltham, MA, USA). RNA concentration and quality were measured using NanoDrop OneC (Thermo Fisher Scientific, USA). Next, 1 μg of total RNA was treated with DNase I (Thermo Fisher Scientific, USA). An MMLV RT kit (Evrogen, Moscow, Russia) was used to synthesise the first strand of cDNA. The resulting cDNA was diluted 1:10 in nuclease-free water and used as a template for the qRT-PCR.

qRT-PCR was performed using a DT-96 PCR machine (DNA-Technology, Moscow, Russia) under the following thermal cycling conditions: initial cycle at 50 °C for 2 min and initial denaturation for 2 min at 95 °C, followed by 50 annealing/elongation cycles (95 °C for 15 s and 60 °C for 60 s). The volume of the reaction mixture for one reaction was 20 µL, which included 4 µL of the cDNA template, 2 µL of a 10 µM mixture of forward and reverse primers, 4 µL of the qRT-PCR mix-HS SYBR reaction mixture (Evrogen, Russia), and 10 µL of nuclease-free water. To calculate changes in gene expression (RQ), we used the ∆∆Ct model [83]. A significant change in gene expression was considered a twofold increase or decrease in expression compared to both controls.

### 4.4. RNA Sequencing and Transcriptomic Analysis

Total RNA was isolated using the GeneJET RNA Purification Kit (Thermo Fisher Scientific, USA) with polyvinylpyrrolidone addition. The quality and purity of isolated RNA were assessed using NanoDrop OneC (Thermo Fisher Scientific, USA) and horizontal gel electrophoresis. cDNA synthesis, library preparation, and sequencing were provided by “Evrogen” (Moscow, Russia). Using a TruSeq mRNA Stranded reagent kit (Illumina, San Diego, CA, USA), poly(A+)-fraction enrichment and random primer cDNA synthesis were performed in 12 RNA samples. The resulting cDNA was used to prepare libraries compatible with Illumina sequencing technology. The quality of the obtained libraries was checked using Fragment Analyzer (Agilent, Santa Clara, CA, USA). Quantitative analysis was performed using the qRT-PCR method. After quality control, the pool of cDNA libraries was sequenced using Illumina NovaSeq 6000 (150 bp, pair-ended). FASTQ files were obtained using bcl2fastq v.2.20 Conversion Software (Illumina, USA). The format for recording the quality data string was Phred 33. As a result, 1,090,239,068 raw reads were obtained.

The transcriptome data analysis was performed following suggestions previously described in [84]. Quality assessment was performed using FastQC v.0.11.9 and MultiQC v.1.10 software. Trimmomatic v.0.40 software was used for trimming using the following parameters: ILLUMINACLIP:TruSeq3-PE.fa:2:30:10; HEADCROP:11; LEADING:3; TRAILING:3; MAXINFO:130:1; MINLEN:50. The trimmed reads were deposited in Sequence Read Archive under accession number PRJNA958217 and used for de novo transcriptome assembly using Trinity v.2.9.1 in the Galaxy public server (https://usegalaxy.eu, accessed from 15 September 2022 to 15 March 2023) [85]. The in silico normalisation of paired and unpaired reads was performed, and unpaired readings were attached to the “left” paired read. Contigs smaller than 200 bp were filtered from further analysis. Assembly completeness was tested in BUSCO v.2 [86]. The fabales_odb10 data set corresponding to representatives of the *Fabaceae* family was used for evaluation. The results showed 89.4% similarity of the assembly with the group of orthologues and a high percentage of duplications (84.0%), which may indicate the possible tetraploidy of the samples since *V. cracca* is known to possess either diploid or autotetraploid cytotypes [87,88]. The alignment of paired reads to the assembled transcriptome showed a satisfactory result (above 60%). Open reading frames were predicted for the assembled contigs in TransDecoder v.5.5.0. The search for protein (blastp) and translation (blastx) motifs were performed on sequences obtained from the Swiss-Prot database. Protein domains were identified in HMMER v.2.2 [89] and Pfam [90]. Predictions of signal peptides, rRNA transcripts, and transmembrane regions were performed in SignalP v.4.1 [91], RNAMMER v.1.2 [92], and TmHMM v.2.0, respectively. Functional annotation was carried out in Trinotate v.3.2.2 [93] and InterProScan 5.59_91.0 [94]. As a result, gene annotations of contigs and terms of gene ontology (GO) were obtained.

The quantification algorithm kallisto v.0.46 [95] and DESeq2 statistics package [96] were used to quantify gene expression. Comparisons were made between contaminated and control plots and two control plots (Babchin–Lomysh) to assess heterogeneity (Figure S10). Genes with log_2_FC ≥ |2| and adjusted *p*-values ≥ 0.05 (Benjamini–Hochberg method) were considered as differentially expressed. The DAVID Bioinformatics tool [97] was used to identify significant GO terms. Venn diagrams were created using the web tool http://bioinformatics.psb.ugent.be/webtools/Venn (accessed on 15 March 2023).

### 4.5. Protein Isolation and Proteomic Analysis

For protein isolation, at each control plot, we used 3 replicates and 4 replicates for each radioactively contaminated plot. Two buffers were used to isolate and purify protein from *V. cracca* leaves: (1) extraction buffer (EB): 0.2 M TRIS-HCl, Ph 9.0; 0.2 M KCl; 0.025 M EGTA, Ph 8.0; 0.035 M MgCl_2_; 1% (*v*/*v*) PTE; 1% (*v*/*v*) detergent blend (Brij-35, Tween 20, Triton X-100, Igepal CA 630); 0.005M DTT; 1× cocktail of protease inhibitors; (2) lysis buffer (LB): 4% SDS; 100 mM Tris/HCl, pH 7.4; 1× cocktail of protease inhibitors. Then, 100 mg of tissue per sample was ground in liquid nitrogen and dissolved in 750 μL of buffer EB on ice for 20 min, pipetting until the complete dissolution of plant tissue aggregates. The suspension was centrifuged at maximum speed for 10 min at 4 °C. The supernatant was collected and stored on ice. The precipitate was redissolved in 500 μL of LB, incubated for 3 min at 95 °C, and then sonicated for 5 min to destroy the membranes. After centrifugation, the supernatants were combined, and 10% TCA in acetone pre-cooled to −20 °C was added to precipitate the proteins. This solution was incubated overnight at −20 °C and then centrifuged for 10 min. The protein precipitate was then washed twice with cold acetone and dried. Before the analysis, the protein pellet was stored at −80 °C.

The Skolkovo Institute of Science and Technology provided proteomic analysis services. The precipitated protein was dissolved in 200 µL of sample dilution buffer, placed in an ultrasonic bath for 1 min, and centrifuged for 10 min at 14,000× *g* and 10 °C. The supernatant was transferred to clean, labelled vials. The following mix was prepared to estimate the amount of protein via the BSA method: 5 µL of the supernatant obtained during lysis, 25 µL of water, 1 mL of the BSA reagent, and 20 µL of a 4% copper sulphate solution. To prepare a blank sample, 30 µL of water, 1 mL of the BSA reagent, and 20 µL of a 4% copper sulphate solution were mixed. Similarly, calibrant samples were prepared (solutions of BSA with a concentration of 1 µg/µL to 10 µg/µL) with BSA reagent. The samples were shaken and incubated for 20 min at 56 °C with stirring on a thermomixer. The samples were cooled to room temperature and analysed on a CLARIOstar reader (BMG Labtech, Ortenberg, Germany). For further analysis, samples were diluted in 50 mM TEAB to a final 2 µg/µL protein concentration.

For protein hydrolysis, 10 µL of samples were transferred into 200 µL tubes, corresponding to 20 µg of protein. Then, 10 µL of protein recovery mixture was added to the samples, shaken, rapidly centrifuged, then incubated for 30 min at 80 °C and cooled to room temperature. Next, 100 µL of 50 mM TEAB and 2 μL of trypsin solution (trypsin:protein ratio 1:100) were added into the samples and incubated for 4 h at 42 °C. Next, another 2 μL of trypsin solution was added (trypsin:protein ratio 1:50), incubated for 18 h (overnight) at 37 °C, and then cooled to room temperature. Formic acid (2 μL) was added, shaken, and cooled for 10 min in a refrigerator at 4 °C, then centrifuged for 10 min at 14,000× *g* and 10 °C. The supernatant was transferred into glass inserts for HPLC-MS analysis and evaporated in a vacuum concentrator, then redissolved in 10 μL of 0.1% formic acid in water. Then, samples were analysed via high-performance liquid chromatography with tandem mass spectrometry (HPLC-MS/MS) using the UltiMate 3000 RSLCnano system coupled with a Q Exactive HF hybrid quadrupole-Orbitrap mass spectrometer (Thermo Scientific, USA). Samples were analysed in three technical replicates. Then, 2 μL per sample was separated on an Acclaim Pepmap C18 column (Thermo Scientific, USA) in a gradient elution mode. The gradient was formed by mobile phase A (0.1% formic acid in deionised water) and mobile phase B: (80% acetonitrile, 0.1% formic acid in deionised water). The column was washed with 2% mobile phase B for 1 min at a flow rate of 700 nL/min, then 1 min at 500 nL/min and 3 min at 400 nL/min. Further separation was carried out at a flow rate of 400 nL/min: the concentration of mobile phase B was linearly increased to 5% in 3 min, and then, the concentration of phase B was linearly increased to 30% in 70 min. Next, the concentration of phase B was linearly increased to 99% in 2 min, and after a 5 min wash in 99% buffer B, the concentration of buffer B was linearly reduced to the initial 2% over 3 min, and the column was washed with 2% mobile phase B for 8 min. The total duration of the analysis was 95 min.

Mass spectrometric analysis was performed on a Q-Exactive HF mass spectrometer in positive ionisation mode using a NESI source (Thermo Scientific, USA). The following parameters were set: emitter voltage: 2.1 kV; capillary temperature: 240 °C. Panoramic scanning was carried out in the mass range from 390 *m*/*z* to 1200 *m*/*z* at a resolution of 60,000. In tandem scanning, the measurement was carried out in the mass range from 200 *m*/*z* to the upper limit, which was determined automatically based on the mass of the precursor, but not more than 2000 *m*/*z*, at a resolution of 15,000. Precursor ions were isolated in a window of ±1 Da. The maximum number of ions allowed for isolation in the MS/MS mode was no more than 10, the minimum intensity of the precursor ion for tandem analysis was set as 50,000 units, and the normalised collision energy was 29. For tandem scanning, only ions from z = 2+ to z = 4+ were in charging conditions. The maximum accumulation time for precursor ions was 50 ms, and for fragment ions, it was 150 ms. All measured precursors were dynamically excluded from the tandem MS/MS analysis for 10 s.

Proteins were identified using the MaxQuant v.1.6.17.0 software and the Andromeda search algorithm. Proteins were identified using the UniProt database (UniProt release 2022_02) with species indication. The following search parameters were set: the cleaving enzyme trypsin, the accuracy of mass determination of *m*/*z* peptides in MS ± 5 ppm, the accuracy of mass determination in MS/MS spectra ± 0.01 Da, and the possibility of skipping two trypsin cleavage sites. Proteins were considered reliably identified if at least two peptides were found. The oxidation of methionine, the acetylation of the N-terminus of the protein, and the modification of cysteine with chloroacetamide were considered possible and mandatory modifications of the peptides. The False Discovery Rate value of no more than 1.0% was used to validate comparisons of Peptide–Spectrum Matches in the spectra and peptides. Label-free protein quantification was based on iBAQ. The data were filtered to remove possible contaminants and unreliably identified proteins, and only proteins that occurred in two technical repetitions of the MS analysis were retained. We only used proteins with log2FC ≥ |1| for further functional evaluation compared to the control samples.

### 4.6. Metabolite Extraction and Metabolomic Analysis

Metabolite extraction, derivatisation, and GC-MS analysis were performed at Institute Jean-Pierre Bourgin (INRAE), as previously described [98]. The freeze-dried samples of *V. cracca* leaves were ground using metal beads and resuspended in 1 mL of frozen (−20 °C) water:acetonitrile:isopropanol (2:3:3) solution, containing ribitol at 4 mg/mL^−1^ and extracted for 10 min at 4 °C via shaking at 1400 rpm in an Eppendorf thermomixer (Eppendorf, Germany). Insoluble material was removed via centrifugation at 20,000× *g* for 5 min. Then, 100 mL of supernatant was collected and dried for 4 h in a Savant Speed-Vac (Thermo Fisher Scientific, USA) and stored at −80 °C. Three blank tubes underwent the same steps as the samples. Quality controls (QCs) were prepared by pooling the same amount of each sample. They were injected at the beginning, the middle, and the end of the injection series to ensure the stability of the derivatisation. After drying for 1 h after −80 °C storage, 10 mL of 20 mg/mL^−1^ methoxyamine in pyridine was added to the samples. The reaction was performed for 90 min at 28 °C under continuous shaking in the Eppendorf thermomixer. Then, 50 mL of N-methyl-N-trimethylsilyl-trifluoroacetamide was added, and the reaction continued for 30 min at 38 °C. After cooling down, 45 mL of the derivatised sample was transferred to an Agilent vial for injection.

Metabolites were analysed via GC-MS 4 h after derivatisation. In total, 1 μL of the derivatised sample was injected in the split and splitless modes in an Agilent 7890A gas chromatograph coupled to an Agilent 5975C mass spectrometer, column Rxi-5SilMS from Restek (30 m with 10 m Integra-Guard column; Bellefonte, PA, USA). The oven temperature ramp was 70 °C for 7 min and then 10 °C/min to 330 °C for 4 min (run-length 36.5 min). The helium constant flow was 0.7 mL/min in the splitless mode and 1 mL/min in the split mode. The temperatures were as follows: injector—250 °C; transfer line—300 °C; source—250 °C, and quadripole—150 °C. Samples were randomised. An alkane mix (C10, C12, C15, C19, C22, C28, C32, and C36) was injected in the middle of the queue for external retention index calibration. Five scans per second were acquired.

Raw Agilent data files were converted to NetCDF format and analysed with AMDIS 32 [99]. A home AMDIS retention index/mass spectra library, built from the NIST, Golm, and Fiehn databases and standard compounds, was used for metabolite identification. Peak areas were determined more precisely using the Target-Lynx software (Waters, France) after converting the NetCDF file to MassLynx format. Metabolites were normalised to the ribitol internal standard and to dry weight. A subset of metabolites was quantified absolutely using a response coefficient in both injection modes to ensure the absence of saturation.

Statistical analysis was carried out with Metaboanalyst 5.0, a web-based metabolomic processing software, using multivariate and hierarchical clustering statistical approaches [100]. If a metabolite was not identified in one of the plots but was presented in others, the missing values were replaced by 1 × 10^−15^. In other cases, the remaining missing values were replaced by a limit of detection (1/5 of the minimum positive value of each variable). Before the statistical analysis, all data were subjected to sample normalisation via median and auto-scaling. Principal component analysis (PCA) and partial least squares–discriminant analysis (PLS-DA) were carried out to determine the metabolomic variability within the experimental plots. Metabolite change measurements were subjected to one-way ANOVA. Significant differences between individual means were determined using Fisher’s least significant difference pairwise comparison test at the 5% confidence level. To visualise the relationship among samples and among the top 50 metabolites, hierarchical clustering heatmaps with Euclidean distances as the similarity measure and Ward’s linkage as the clustering algorithm were conducted. Volcano plots were also generated for all detected metabolites, and the significant metabolites with *p* ≤ 0.05 and FC ≥ 2 were highlighted based on Student’s *t*-test. Significant metabolites were used for the pathway analysis, combining powerful pathway enrichment analysis results with pathway topology analysis to identify the most relevant pathways involved in the plant response to chronic radiation exposure. The *Arabidopsis thaliana* pathway library was used as a reference set.

### 4.7. Multi-Omics Data Integration

Multi-omics analysis was conducted with the web tool PaintOmics 4 [101]. The closest to *V. cracca* model species in KEGG databases, *Medicago truncatula* L., was used as a proxy for multi-omics analysis. The Galaxy public server (https://usegalaxy.eu/, accessed from 15 September 2022 to 15 March 2023) [85] was used to perform a blastp search of the differentially expressed *V. cracca* transcripts and proteins against the *M. truncatula* proteome (retrieved from the Uniprot database, entry UP000002051). The E-value cutoff was set as 0.001, and the other parameters were used as default. RStudio v.4.2.2 was used to prepare data for the multi-omics analysis. Background data on transcriptomics, proteomics, and metabolomics were provided for PaintOmics 4 software as log_2_FC in irradiated samples compared to controls (pairs K × B, K × L, M × B, and M × L). From metabolomic data, log_2_FC was only provided for those metabolites with KEGG ID. As relevant features for the PaintOmics 4 tool, we used differentially expressed genes (DEGs) and differentially expressed proteins in both contaminated plots. As relevant metabolites, we chose those significantly different from the control for at least one experimental plot. After selecting and preparing datasets, the following parameters were used to run PaintOmics4: species: *Medicago truncatula* L.; databases: KEGG, MapMan, and Reactome; clusters presented in data: generate automatically; metabolite class activity threshold: generate automatically.

## 5. Conclusions

*Vicia cracca* plants growing in radioactively contaminated plots under multiple generations of chronic radiation exposure were characterised by complex and multidirectional changes in the molecular phenotype. Exposed plants had significant alterations in their metabolism and gene expression patterns compared with plants from two reference plots. At the level of carbon metabolism, the upregulation of glycolate, pyruvate, and fumarate biosynthesis pathways and the downregulation of citrate synthesis suggests that the cells are trying to bypass some steps of the TCA cycle to generate key metabolic intermediates. At the level of energy metabolism, the downregulation of glycolysis pathways, alterations in electron transport chain components, and changes in the photosynthetic machinery support a decrease in the cellular ability to generate ATP. The downregulation of fatty acid biosynthesis pathways and alterations in the glyoxylate cycle indicate a shift in the cellular metabolism away from fatty acid biosynthesis and towards using odd-chain fatty acids or amino acids as alternative energy sources. Profound changes in carbon metabolism may indicate the reallocation of carbon skeletons for secondary metabolite production to increase the antioxidant capacity of plants. Those effects were accompanied by the suppression of ribosomal biogenesis and translation. Overall, the changes observed in carbon metabolism, energy metabolism, nitrogen metabolism, and gene expression suggest a complex biochemical response of *Vicia cracca* plants in the Chernobyl exclusion zone.

Moreover, chronically irradiated plants showed signs of redox imbalance and the activation of protective metabolic pathways. The upregulation of histones and few DNA repair machinery genes suggests that plants probably rely on DNA packaging and homologous recombination to cope with chronic DNA damage. Yet, it is still unknown if the observed changes would be linked to strand break repair or DNA replication errors. The upregulation of peroxidases and genes involved in phenolic compound biosynthesis suggests increased ROS concentrations and a compensatory increase in antioxidant production. The nitrogen redirection towards the biosynthesis of secondary metabolites and profound changes in carbon metabolism indicate resource reallocation to cope with environmental conditions. The changes in ribosomal protein synthesis and RNA polymerases further suggest that the plant undergoes significant gene expression and translational pattern alterations.

Several molecules can be promising as radiation exposure biomarkers and for testing the resilience of *Vicia* plants to other stressors. Among them are chaperonin CPN60A, histones, including H2B and H4; transcription factor HY5; and chlorophyll-binding protein CAB1, as their genes show consistent upregulation for different plant species growing under chronic radiation exposure. The expression of the phenylalanine ammonia-lyase (PAL) gene and the abundance of its protein can be used to approximately assess the activation of secondary metabolites’ biosynthesis under chronic irradiation. Metabolites that seem responsive to chronic irradiation include the TCA components such as citrate, pyruvate, malonate, fumarate, and various inositol metabolites and RFOs. Especially interesting for future research is the involvement of sugar signalling in photosynthesis suppression under radiation exposure and the role of certain ribosomal proteins as a possible source for radiosensitivity manipulations. Future research could also explore the molecular mechanisms (probably epigenetic) underlying the observed changes in DNA repair machinery and histone expression.

In conclusion, the study of the multi-omic responses of *V. cracca* to chronic radiation exposure highlights its ability to adapt to stressful environmental conditions through complex and multidirectional changes in gene expression and metabolism. Comparing the responses of different plant species to chronic radiation exposure could reveal universal stress-tolerant mechanisms that could be manipulated to improve the resilience of crops. The findings of this study have implications for agricultural practices and ecosystem management strategies, as well as for the development of stress-tolerant crops.

## Figures and Tables

**Table 1:** Radioactive contamination of experimental plots.

Experimental Plots	Plot Type	GPS Coordinates	Ambient Dose Rate (γ) μSv × h^−1^	α-Particles Flux Density min^−1^ × sm^−2^	β-Particles Flux Density min^−1^ × sm^−2^	Specific Activity of ^137^Cs in Soil Bq × kg^−1^	Specific Activity of ^90^Sr in Soil Bq × kg^−1^
Babchin (Bab, B)	Reference	N 51°47′29.43″ E 30°00′03.86″	0.29	0.4	4.7	126 ± 4.5	9.86 ± 1.6
Lomysh (Lom, L)	Reference	N 51°49′50.56″ E 29°34′37.03″	0.35	2.5	2.4	1027 ± 30	16.09 ± 2.6
Kulazhin (Kul, K)	Polluted	N 51°33′07.6″ E 30°13′10.5″	6.84	42.0	31.0	36,990 ± 935	1396.6 ± 209.8
Masany (Mas, M)	Polluted	N 51°30′22.36″ E 30°00′56.86″	3.20	38.7	47.2	9587 ± 246	1598.5 ± 240.1

**Table 2:** Soil properties in the experimental plots.

Experimental Plots	pH	HA	TEB	Humus	Av. P_2_O_5_	Av. K_2_O	Av. Ca	Av. Mg	Av. Na
		mg-eqv Per 100		%	mg/kg		mg-eqv Per 100 g		
Babchin (Bab, B)	6.4	0.90 ± 0.01	6.1 ± 0.7	1.17 ± 0.01	210.7 ± 5.2	91.0 ± 1.0	3.41 ± 0.20	0.85 ± 0.02	0.61 ± 0.04
Lomysh (Lom, L)	5.5	1.58 ± 0.02	3.1 ± 0.1	1.78 ± 0.04	209.1 ± 5.2	38.7 ± 0.1	2.49 ± 0.08	0.53 ± 0.01	0.57 ± 0.02
Kulazhin (Kul, K)	4.6	7.04 ± 0.07	6.2 ± 0.4	5.28 ± 0.07	337.1 ± 6.4	47.5 ± 0.7	5.13 ± 0.01	2.09 ± 0.02	1.18 ± 0.14
Masany (Mas, M)	5.5	3.71 ± 0.08	7.6 ± 0.2	2.67 ± 0.02	943.2 ± 19.9	153.5 ± 2.6	5.06 ± 0.19	0.79 ± 0.01	0.64 ± 0.05

**Note:** HA—hydrolytic acidity; TEB—total exchangeable bases; Av.—available.

**Table 3:** GO terms for shared DEGs from both radioactively contaminated plots compared to both controls.

Upregulated		Downregulated	
GO:0000786	nucleosome	GO:0009793	embryo development ending in seed dormancy
GO:0003677	DNA binding	GO:0009534	chloroplast thylakoid
GO:0046982	protein heterodimerisation activity	GO:0009506	plasmodesma
		GO:0009507	chloroplast
		GO:0009941	chloroplast envelope
		GO:0009570	chloroplast stroma
		GO:0009536	plastid
		GO:0009706	chloroplast inner membrane
		GO:0000325	plant-type vacuole
		GO:0003729	mRNA binding

**Table 4:** Fifty most modulated genes in both experimental plots.

**DOWNREGULATED GENES**						
**Uniprot ID**	**Physiological Process**	**Description**	**Log_2_FC**			
			**B**		**L**	
			**M**	**K**	**M**	**K**
TKTC_CRAPL	Photosynthesis, the Calvin cycle	Transketolase, chloroplastic	−11.6	−9.8	−10.4	−10.4
RBS_MEDSA		Ribulose bisphosphate carboxylase small subunit, chloroplastic	−11.5	−11.7	−9.8	−9.7
KPPR_MESCR		Phosphoribulokinase, chloroplastic	−10.6	−9.8	−9.1	−9.9
RCA1_LARTR		Ribulose bisphosphate carboxylase/oxygenase activase 1, chloroplastic	−10.1	−9.2	−10.1	−9.0
GLGS2_VICFA		Glucose-1-phosphate adenylyltransferase small subunit 2, chloroplastic	−10.0	−10.6	−6.7	−6.3
PGL1B_ARATH	Photosynthesis, light-dependent reactions	Ferredoxin-plastoquinone reductase	−10.6	−10.7	−9.1	−9.2
FTSI1_ARATH	Chlorophyll biosynthesis	FtsH extracellular protease family	−10.1	−10.1	−6.9	−7.0
CHLM_ARATH		Magnesium-protoporphyrin IX methyltransferase	−10.1	−10.1	−6.9	−7.0
CHLP_ARATH		Pyridine nucleotide-disulfide oxidoreductase family protein	−10.8	−10.8	−7.4	−7.6
THI4_CITSI	Thiamine biosynthesis	Thiamine thiazole synthase, chloroplastic-like	−11.0	−10.9	−8.1	−8.3
PUR5_VIGUN		Phosphoribosylformylglycinamidine cyclo-ligase, chloroplastic/mitochondrial	−10.7	−10.6	−7.0	−7.1
SYWM_ARATH	Translation	Nucleotidylyl transferase superfamily protein	−10.6	−10.5	−7.3	−7.4
RS6_ASPOF		40S ribosomal protein S6	−10.1	−10.3	−8.4	−8.6
IF5A_SENVE		Eukaryotic translation initiation factor 5A	−10.2	−10.2	−8.1	−8.3
HSP11_PEA	Stress response	18.1 kDa class I heat shock protein	−10.9	−10.9	−8.8	−9.0
STEP1_ARATH		Stress-enhanced protein 1	−10.5	−11.0	−6.5	−7.1
ROC5_NICSY	RNA processing	33 kDa ribonucleoprotein, chloroplastic	−11.2	−11.1	−8.4	−8.9
STR9_ARATH	Sulphur metabolism	Rhodanese-like domain-containing protein 9, chloroplastic	−11.2	−11.1	−6.9	−7.1
ISPE_SOLLC	Secondary metabolism	4-diphosphocytidyl-2-C-methyl-D-erythritol kinase, chloroplastic/chromoplastic	−10.9	−8.0	−9.0	−6.5
LRX4_ARATH	Cell wall	Leucine-rich repeat (LRR) family protein	−10.9	−10.8	−7.0	−7.2
SNAT2_ORYSJ	Melatonin biosynthesis	Serotonin N-acetyltransferase 2, chloroplastic-like	−10.7	−8.6	−7.1	−6.9
CP31A_ARATH	Telomere elongation	31-kDa RNA-binding protein	−10.7	−11.6	−9.3	−9.6
PGKH_WHEAT	Glycolysis	Phosphoglycerate kinase, chloroplastic-like	−10.3	−10.2	−8.6	−8.8
FT_ARATH	Flowering	Protein FLOWERING LOCUS T	−10.1	−9.2	−9.1	−8.9
CNIH4_ARATH	Vesicle-mediated transport	Cornichon family protein	−10.0	−9.9	−7.8	−7.9
**UPREGULATED GENES**						
**Uniprot ID**	**Physiological process**	**Description**	**Log_2_FC**			
			**B**		**L**	
			**M**	**K**	**M**	**K**
CEP1_ARATH	Programmed cell death	Cysteine proteinases superfamily protein	10.3	7.1	9.3	7.1
PER72_ARATH	Antioxidant system	Peroxidase superfamily protein	9.6	6.0	7.4	6.0
PERE5_ARMRU		Peroxidase E5	9.1	6.4	8.2	6.5
LECS_VATGU	Defence response	Seed lectin	9.4	7.7	8.7	7.7
GUN4C_ARATH	Chlorophyll synthesis	Tetrapyrrole-binding protein, chloroplastic	9.2	8.2	7.6	8.2
ASPG_LUPAL	Protein catabolism	Isoaspartyl peptidase/L-asparaginase	9.4	5.8	7.3	5.8
UBP21_ARATH		Ubiquitin-specific protease 21	9.4	6.0	8.5	6.0
RS31_CANAL		Ubiquitin-ribosomal 40S subunit protein S31 fusion protein	8.8	6.9	8.7	6.9
UFSP_ORYSJ		Probable Ufm1-specific protease	8.7	5.2	5.4	5.2
FB330_ARATH		F-box protein	10.1	8.6	9.1	8.6
TI10A_ORYSJ	Phytohormones	Protein TIFY 10a-like (*jasmonate response*)	8.8	6.6	5.7	6.7
AHP2_ARATH		Histidine-containing phosphotransmitter 2 (*cytokinin response*)	8.8	5.9	6.2	5.9
ILL1_ORYSI		IAA-amino acid hydrolase ILR1-like 1 (*auxin response*)	8.7	6.1	5.6	6.1
XRN2_ARATH	RNA processing	Exoribonuclease 2	9.6	6.8	7.2	6.8
C3H54_ORYSJ		Zinc finger CCCH domain-containing protein 54-like	8.7	7.8	8.7	7.8
PP182_ARATH		Pentatricopeptide repeat (PPR) superfamily protein	8.6	6.6	6.4	6.6
GLSN_MEDSA	Nitrogen assimilation	Glutamate synthase (NADH), amyloplastic	9.0	6.6	6.0	6.6
LHCA2_ARATH	Chloroplast biogenesis	Photosystem I light-harvesting complex protein	9.0	6.5	6.6	6.5
IQD32_ARATH	Transport	IQ-domain 32	8.9	5.9	6.5	5.9
PITC_DICDI		Phosphatidylinositol transfer protein 3	8.8	5.9	8.0	5.9
MED7A_ARATH	Transcription	Mediator complex, subunit Med7(AT5G03220)	8.9	6.7	7.1	6.7
H4_SOYBN	Histone	Histone H4	8.8	6.8	5.9	6.8
PALY_MEDSA	Secondary metabolism	Phenylalanine ammonia-lyase	8.8	7.2	9.6	7.3
TOP3A_ARATH	Cell cycle	Topoisomerase 3-alpha	9.5	6.6	7.2	6.6
CCNB1_MEDSA		G2/mitotic-specific cyclin-1	8.6	8.2	8.8	8.2

**Table 5:** Metabolites with significantly different concentrations in samples from control (**L + B**) and contaminated plots (**K**, **M**).

Metabolite	F Value (ANOVA)	FDR	Fisher’s LSD
Allantoin	55.705	5.84 × 10^−4^	Mas > Control; Mas > Kul
O-methyl-inositol	21.045	4.05 × 10^−2^	Mas > Control; Mas > Kul
Citrate	20.873	4.05 × 10^−2^	Control > Kul; Control > Mas
Inositol-3-phosphate	18.018	5.72 × 10^−2^	Mas > Control; Mas > Kul
Fumarate	17.492	5.72 × 10^−2^	Mas > Control; Mas > Kul
Hydrangeifolin	16.433	6.39 × 10^−2^	Kul > Control; Kul > Mas
Tyrosine	14.835	8.76 × 10^−2^	Kul > Control; Kul > Mas
Raffinose	13.806	9.28 × 10^−2^	Control > Kul; Control > Mas
Maltose	13.706	9.28 × 10^−2^	Kul > Control; Kul > Mas
Pinitol	13.519	9.28 × 10^−2^	Kul > Control; Mas > Control; Mas > Kul
Arabinose	12.781	1.07 × 10^−1^	Kul > Control; Kul > Mas
Malonate	12.547	1.07 × 10^−1^	Mas > Control; Mas > Kul
Benzyl glucopyranoside	11.172	0.016	Kul > Control; Kul > Mas
Pyruvate	9.4194	0.029	Kul > Control; Kul > Mas
Nicotinate	8.5022	0.039	Kul > Control; Mas > Control
Lactitol	8.4345	0.039	Kul > Control; Mas > Control
Phenethyl glycosides	8.2561	0.040	Kul > Control; Kul > Mas
Ribose	7.9636	0.043	Kul > Control; Kul > Mas
Galactosylglycerol	7.4886	0.049	Mas > Control; Mas > Kul
Galactinol	7.4159	0.049	Control > Kul; Control > Mas

**Table 6:** Genes for qRT-PCR analysis.

Gene (*Arabidopsis thaliana*)	AGI	Protein	Function
*APX1*	*AT1G07890*	Cytosolic ascorbate peroxidase APX1	Antioxidant
*CAB1*	*AT1G29930*	Light-harvesting complex subunit II (LHCII)	Photosynthesis
*RBOH-F*	*AT1G64060*	Respiratory burst oxidase homologue F	Response to ABA and ROS
*Hy-5*	*AT5G11260*	Transcription factor HY5	
*PIP1*	*AT3G61430*	Plasma membrane intrinsic protein 1	Aquaporin
*TIP1*	*AT2G36830*	Tonoplast intrinsic protein 1	
*CPN60A*	*AT2G28000*	Chaperonin 60-α	Protein folding
*CPN20*	*AT5G20720*	Chloroplast chaperonin 20	
*H2B*	*AT5G22880*	Histone 2B	Nucleosome assembly

## Data Availability

The processed sequencing files are available at the Sequence Read Archive (BioProject PRJNA958217). Proteomic and metabolomic data before statistical processing and filtering can be found in Tables S3 and S4.

## Supplementary Materials

The following supporting information can be downloaded at: https://www.mdpi.com/article/10.3390/plants12122318/s1, Figure S1: Metabolome, scores; Figure S2: Carbon metabolism; Figure S3: Fatty acid biosynthesis; Figure S4: N-glycan biosynthesis; Figure S5: Glyoxylate and dicarboxylate metabolism; Figure S6: Biotin metabolism; Figure S7: Propanoate metabolism; Figure S8: Ribosome; Figure S9: RNA polymerase; Figure S10: Transcriptome, sample-to-sample distance; Table S1: Sampling and soil properties; Table S2: Differential gene expression; Table S3: Proteomic results; Table S4: Metabolomic results; Table S5: Primer sequences. Reference [82] has been cited in the Supplementary Materials.
