# Supplementary material for: Multi-Omics Analysis of Vicia cracca Responses to Chronic Radiation Exposure in the Chernobyl Exclusion Zone
Source: Plants (Basel). 2023 Jun 14;12(12):2318. doi: 10.3390/plants12122318 (PMC10300722; doi:10.3390/plants12122318)
Supplement: Supplementary file 1 [file plants-12-02318-s001.zip › Table S5 Primer sequences.docx]

Primer pairs for *V. cracca* used in RT-qPCR experiment

| ***Vicia cracca*** | | | |
| --- | --- | --- | --- |
| Gene name (for *A. thaliana*) | Primers for homologue | | bp |
| *APX1* | *f-* ATGGCACTCTGCTGGAACTT  *r-* GGCTCCAAAAGCCTAACAGC | *129* | |
| *CIPK20* | *f-* CAGGCCAAAGTGTTGCCATC  *r-* GCATCACCGAGATTTCACGC | *93* | |
| *CAB1* | *f-* GGTGAGGCTGTGTGGTTCAA  *r-* CAAGATAACCTGGGTGGCCC | *126* | |
| *RBOH-F* | *f-* GTGTCTCCCTTTGAGTGGCA  *r-* TCCCTAACACAGGAGGCTCA | *145* | |
| *SnRK2.4* | *f-* CCTGCCCCTCGTCTGAAAAT  *r-* GGTGCAATGTAGGCTGGAGT | *101* | |
| *TIP1* | *f-* CTTCGGTGCTTTCGTTGGTG  *r-* CTGCGGATAGACCGAATGCT | *143* | |
| *PIP1* | *f-* CACAGGCATCAACCCAGCTA  *r-* ATGAAGGGGCCAACCCAAAA | *99* | |
| *Hy-5* | *f-* GACGGTCCTCAAGTGAAGGG  *r-* TCTTCATCCCATTGCCACCC | *113* | |
| *CPN60А* | *f-* AAAAGGCACTGGTAGCACCT  *r-* ACCAACCTCCCATTCACCATT | *101* | |
| *CPN20* | *f-* AGTCAGTTGGGAAGAGCAAAGT  *r-* TGAACTCCACCTCAGTCCCT | *90* | |
| *H2B* | *f-* ACATCTTCGAGAAGCTCGCC  *r-* TTGGTACCCTCAGAAACGGC | *137* | |
| *ELF1A*  [82] | *f-* GTGAAGCCCGGTATGCTTGT  *r-* CTTGAGATCCTTGACTGCAACATT | 150 | |
| *CYP2*  [82] | *f-* TGCCGATGTCACTCCCAGAA  *r-* CAGCGAACTTGGAACCGTAGA | 200 | |

*
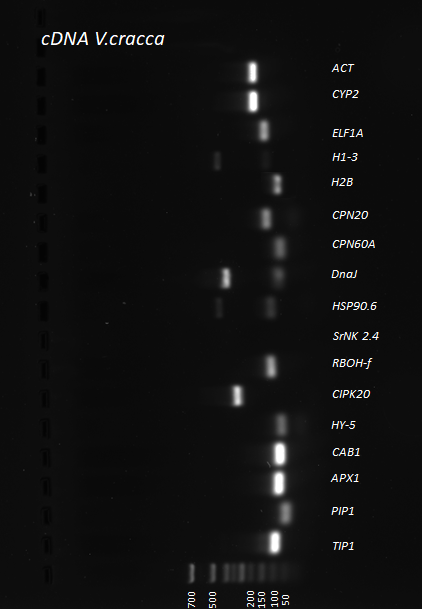
*
