# Supplementary figures and images for: Multi-Omics Analysis of Vicia cracca Responses to Chronic Radiation Exposure in the Chernobyl Exclusion Zone
Source: Plants (Basel). 2023 Jun 14;12(12):2318. doi: 10.3390/plants12122318 (PMC10300722; doi:10.3390/plants12122318)

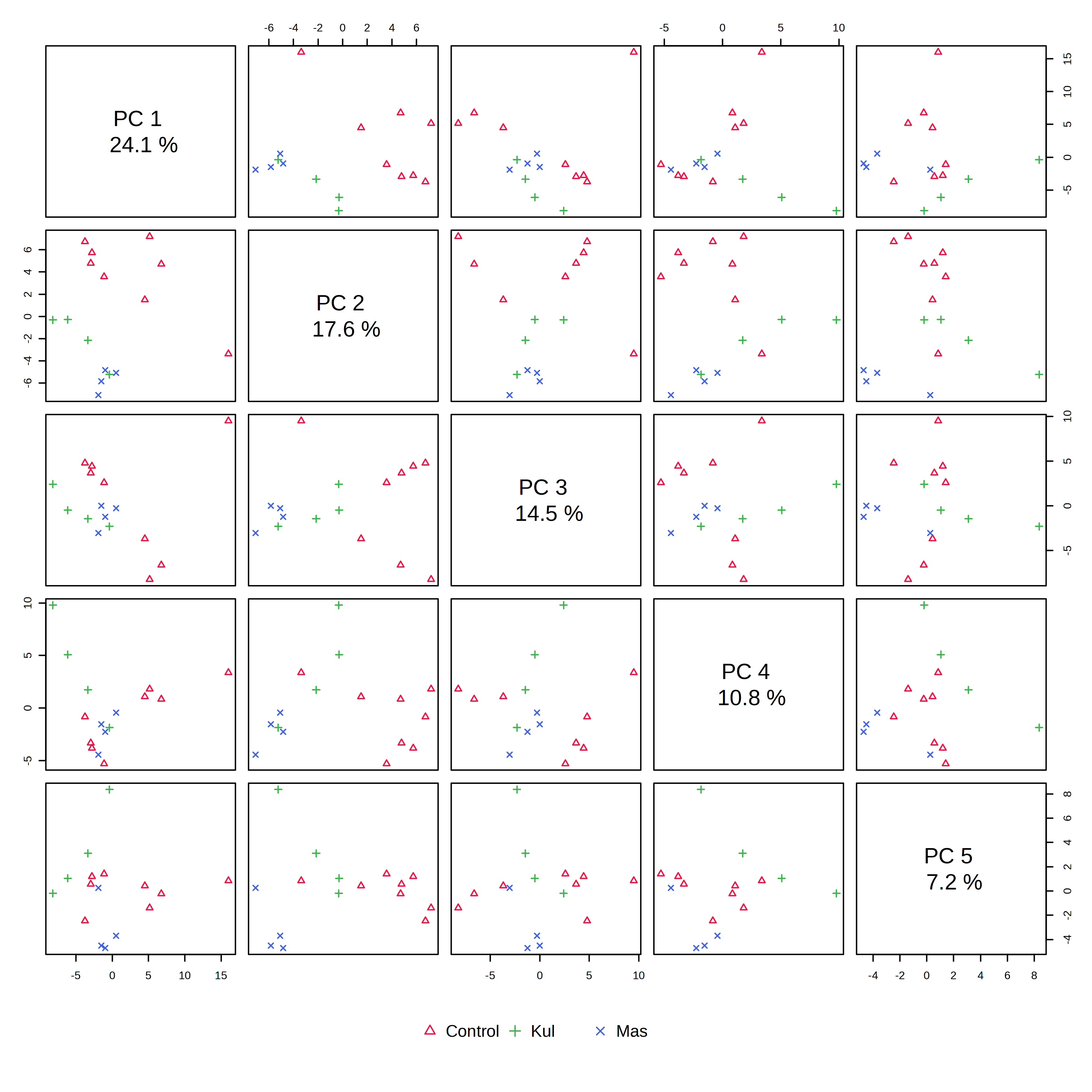

Supplement: Supplementary file 1 [file plants-12-02318-s001.zip › Figure S1 Metabolome, scores.png]

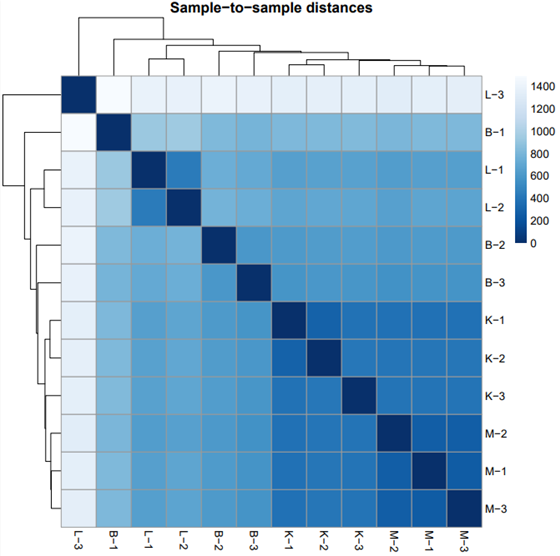

Supplement: Supplementary file 1 [file plants-12-02318-s001.zip › Figure S10 Transcriptome, sample-to-sample distance.png]

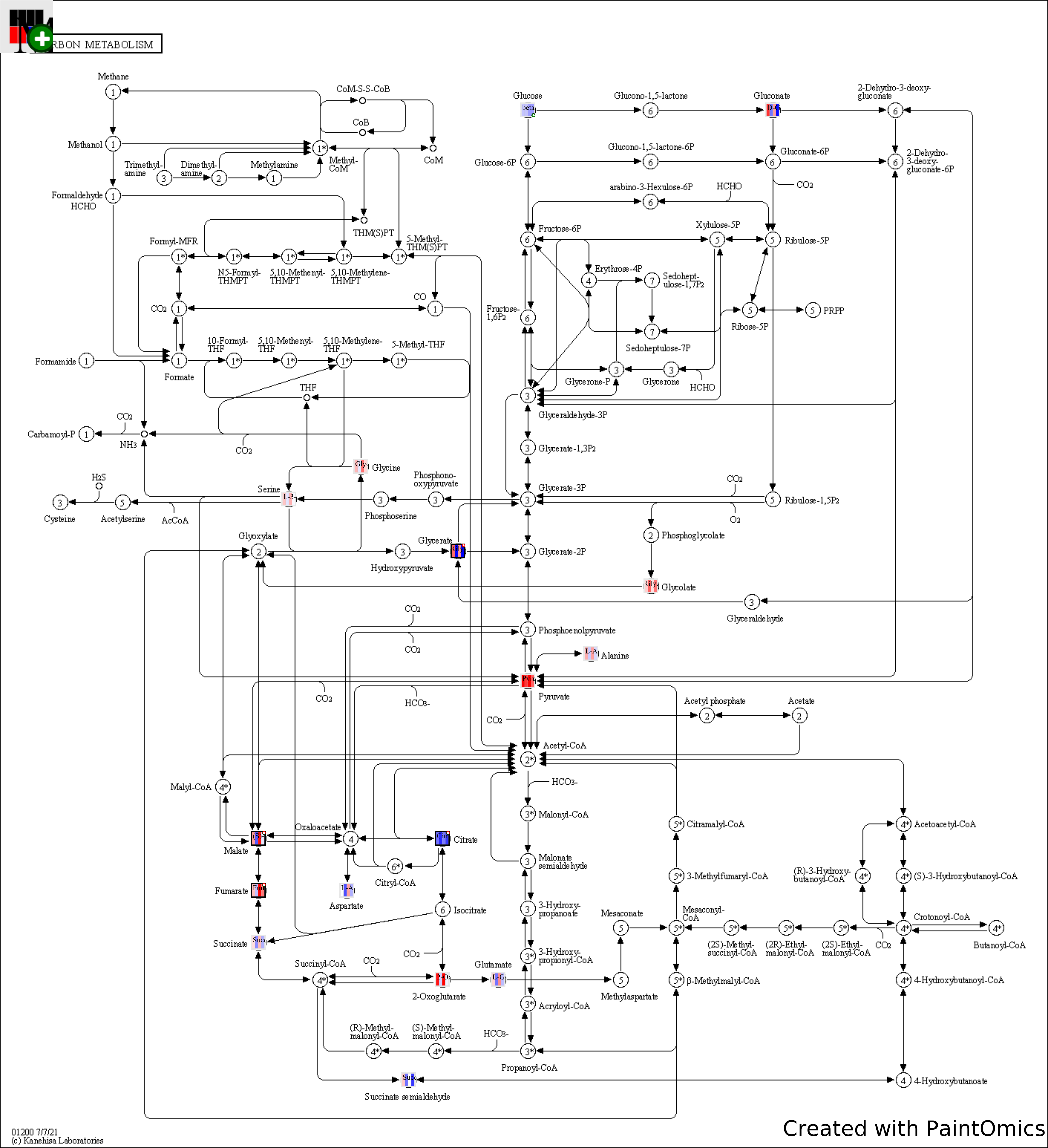

Supplement: Supplementary file 1 [file plants-12-02318-s001.zip › Figure S2 Carbon metabolism.png]

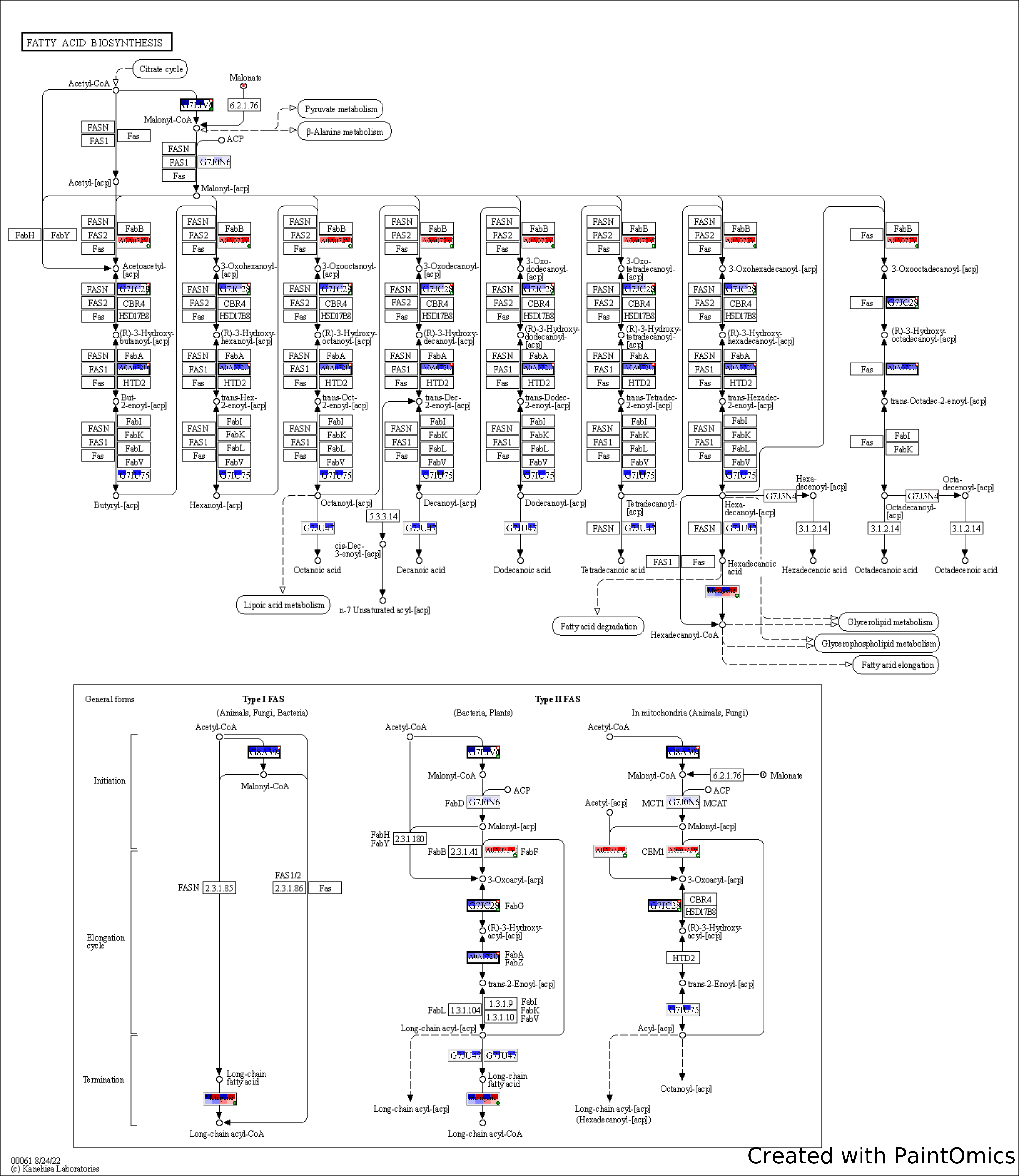

Supplement: Supplementary file 1 [file plants-12-02318-s001.zip › Figure S3 Fatty acid biosynthesis.png]

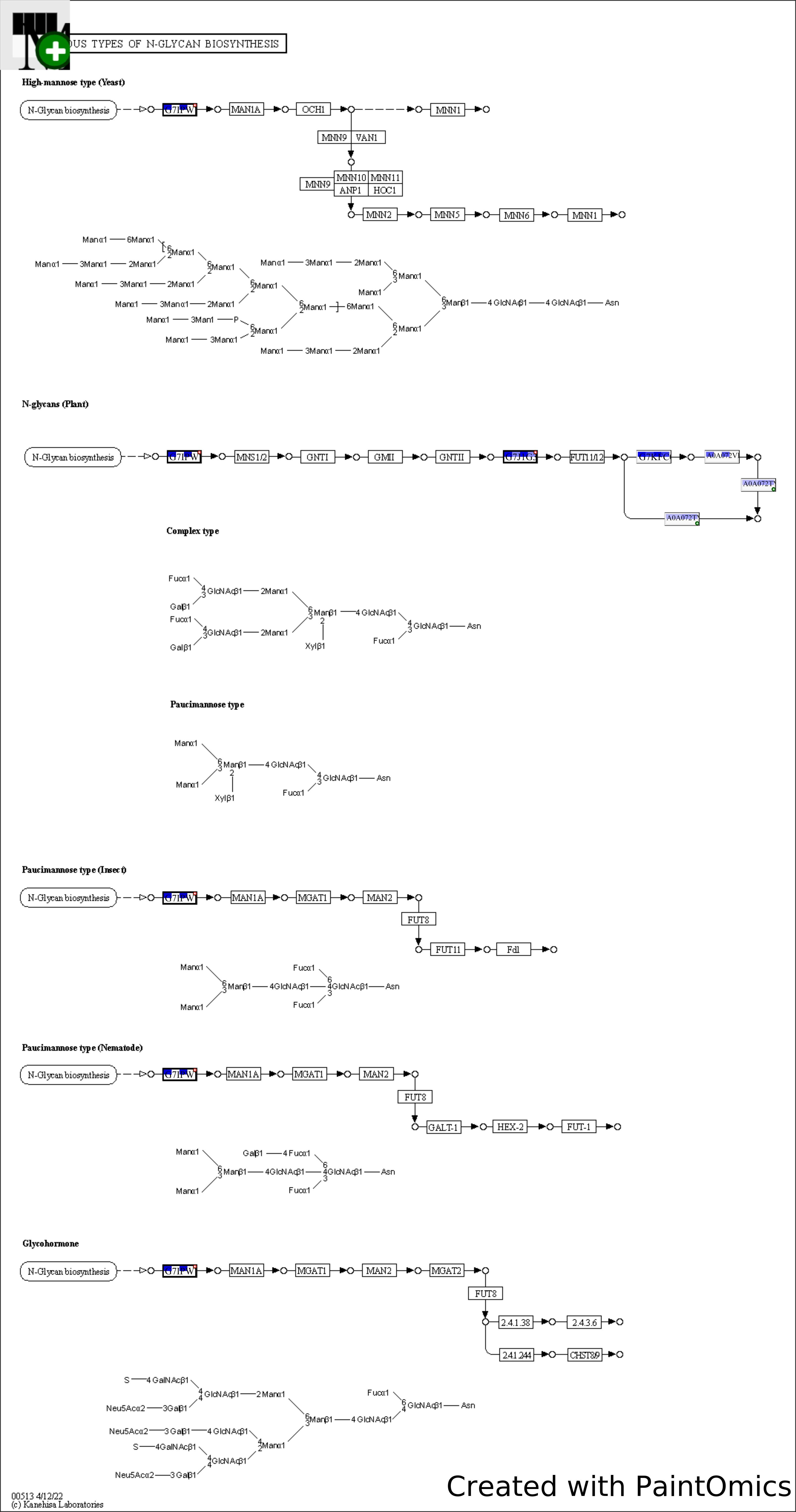

Supplement: Supplementary file 1 [file plants-12-02318-s001.zip › Figure S4 N-glycans biosynthesis.png]

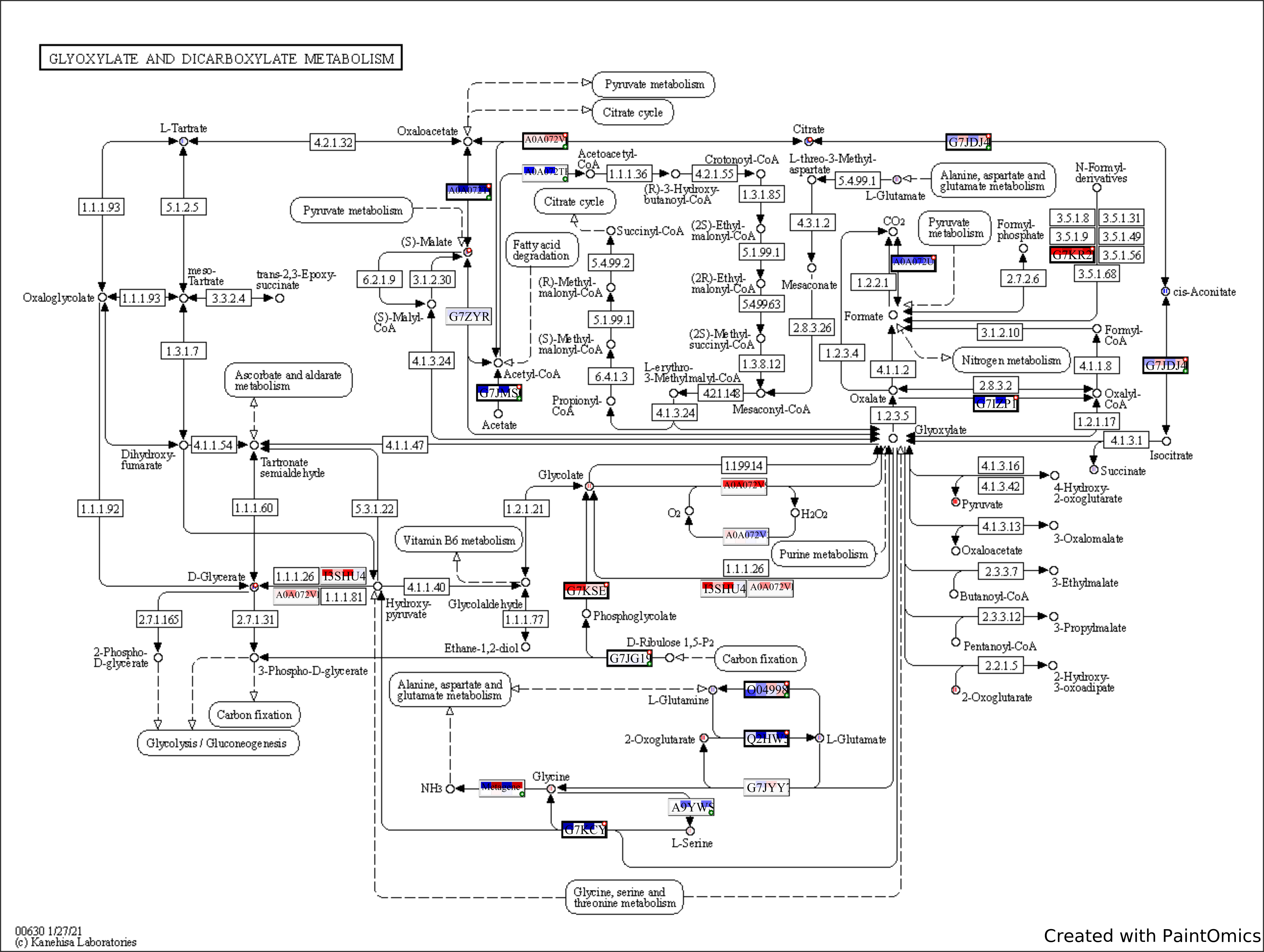

Supplement: Supplementary file 1 [file plants-12-02318-s001.zip › Figure S5 Glyoxylate and dicarboxylate metabolism.png]

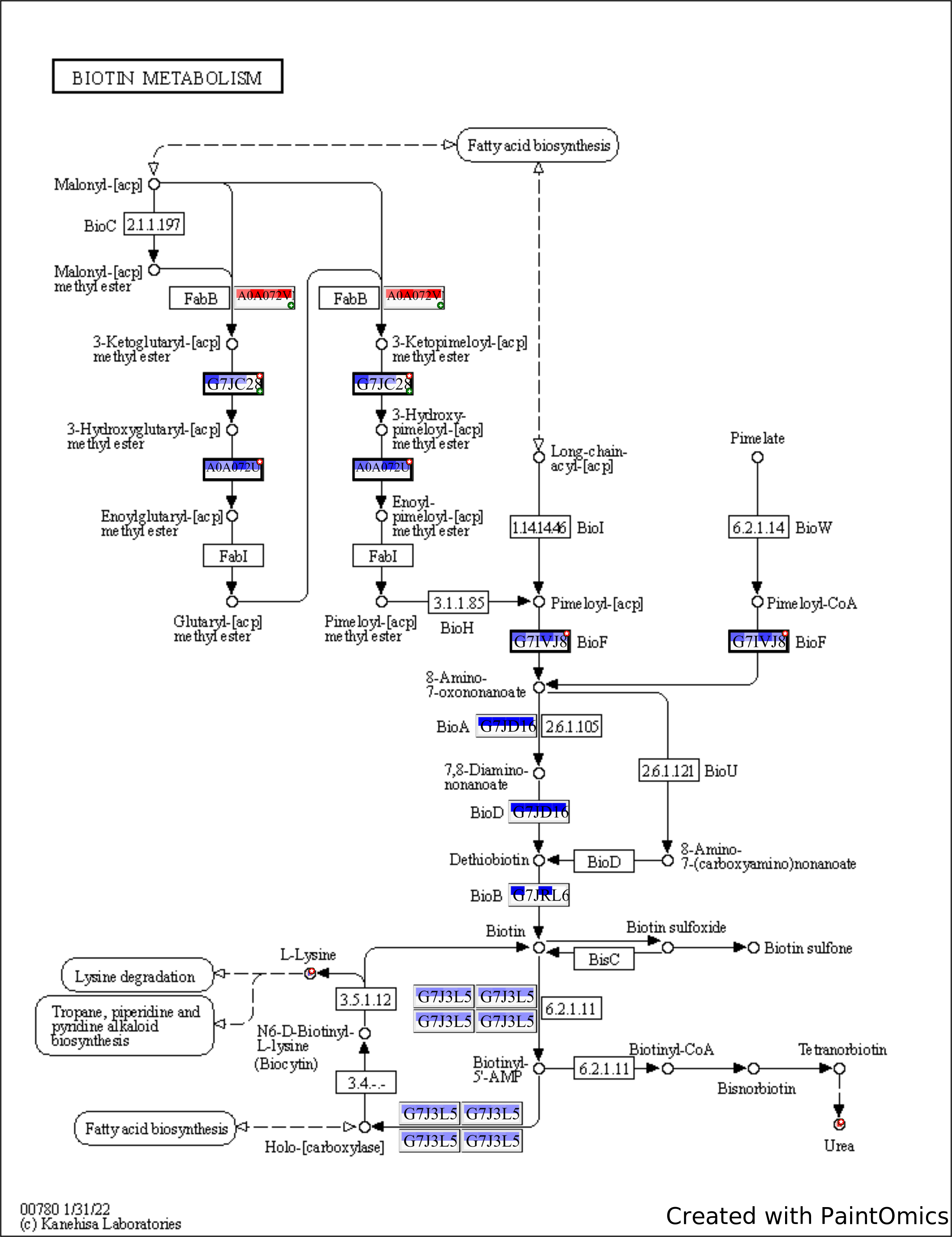

Supplement: Supplementary file 1 [file plants-12-02318-s001.zip › Figure S6 Biotin metabolism.png]

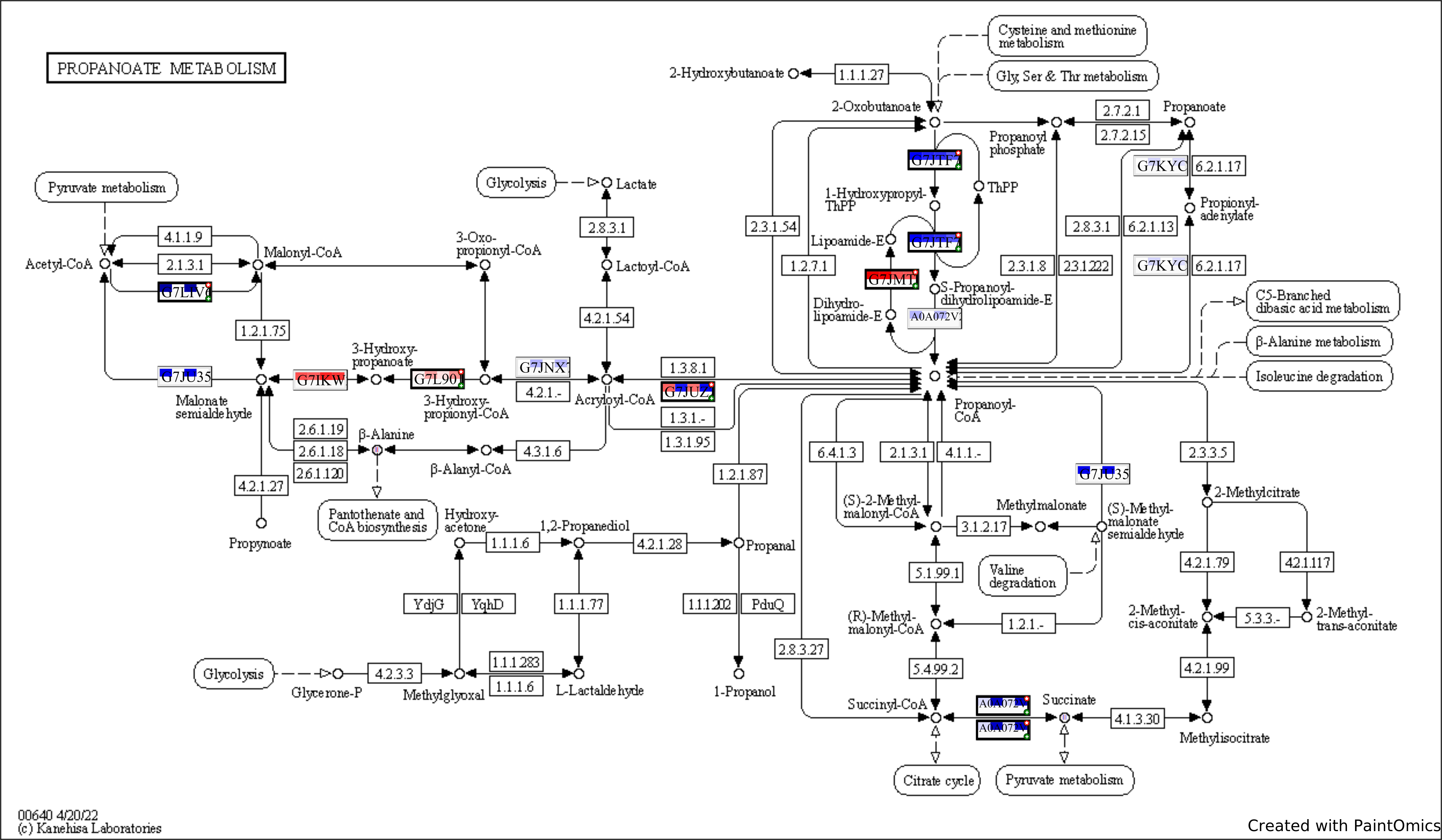

Supplement: Supplementary file 1 [file plants-12-02318-s001.zip › Figure S7 Propanoate metabolism.png]

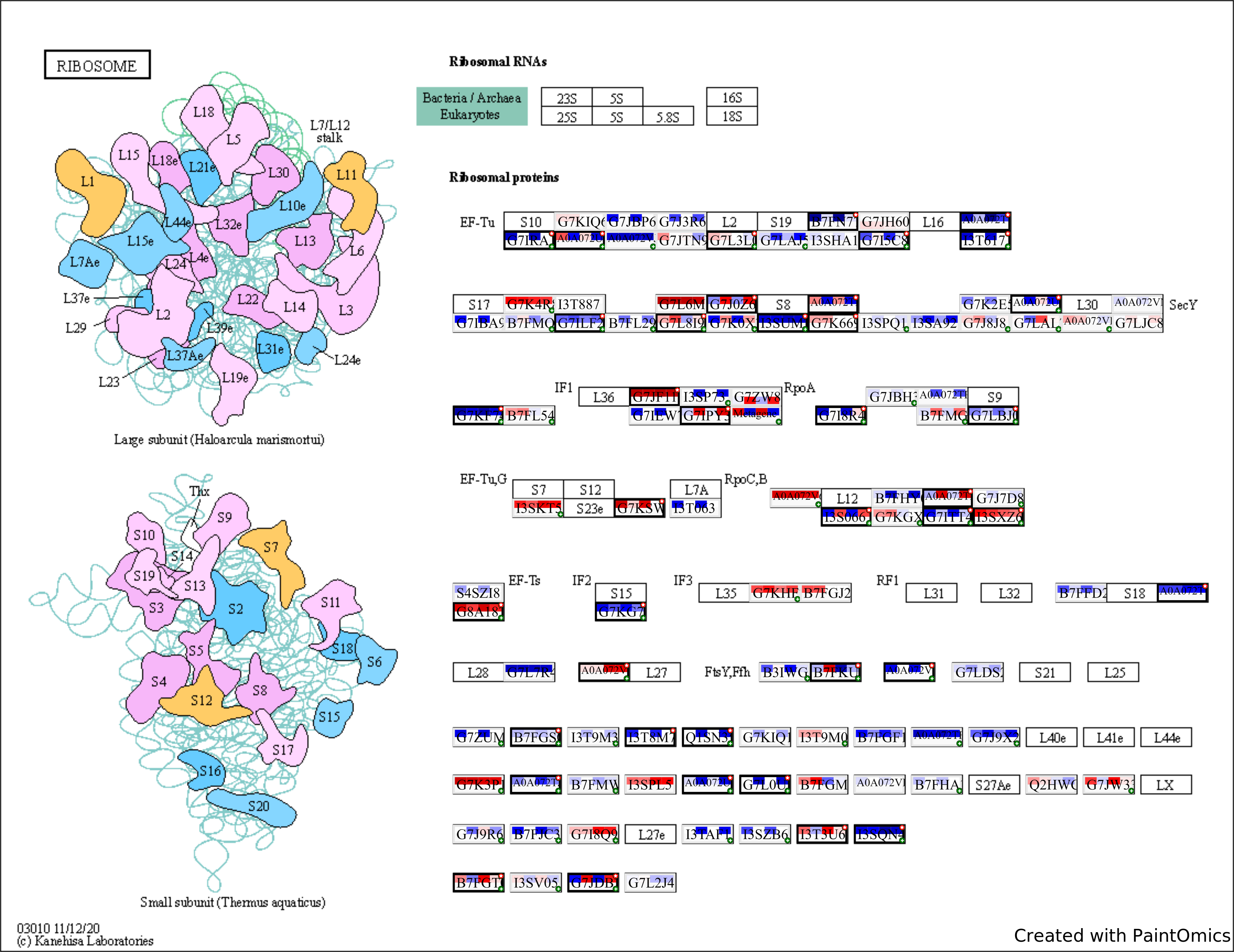

Supplement: Supplementary file 1 [file plants-12-02318-s001.zip › Figure S8 Ribosome.png]

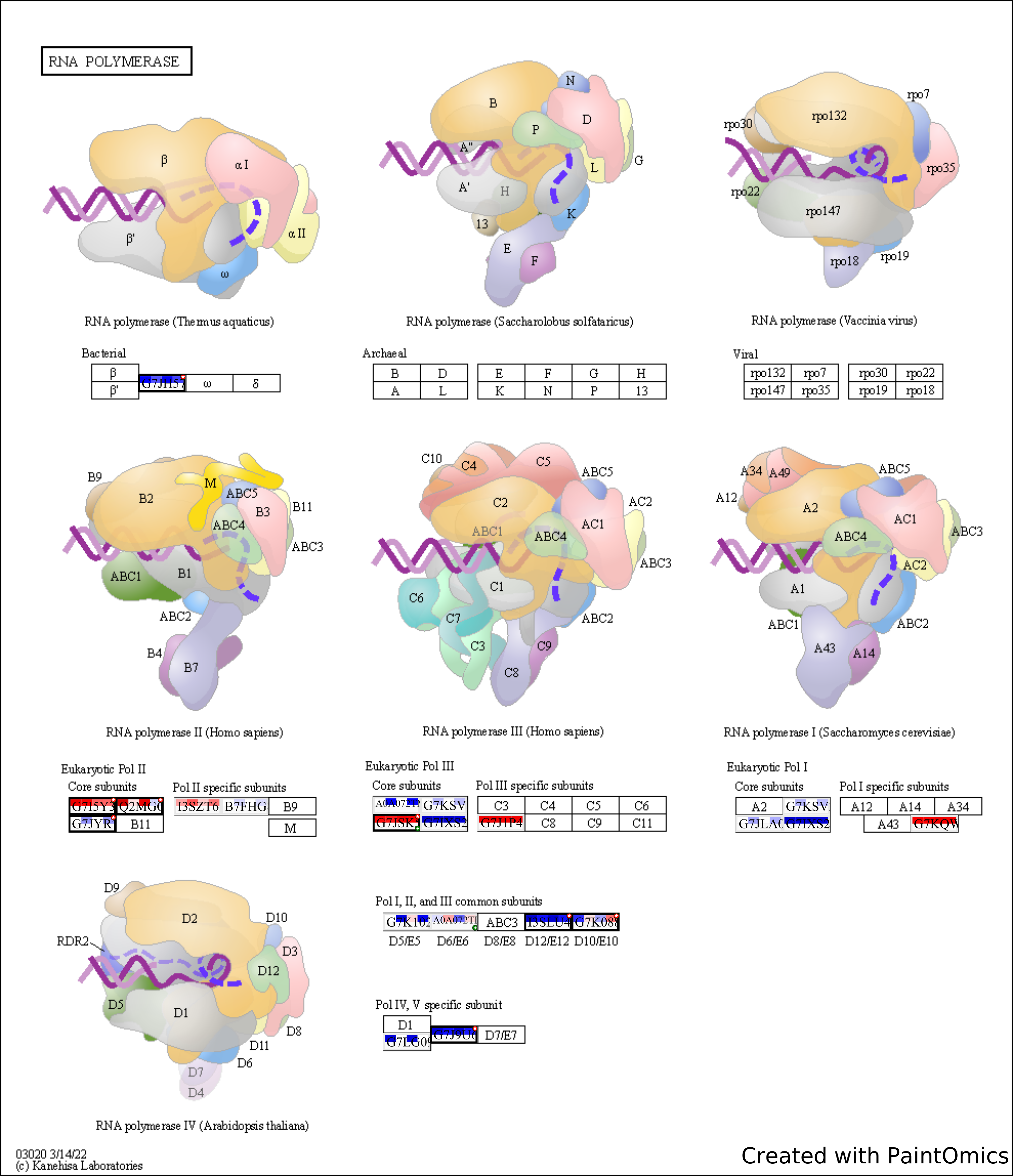

Supplement: Supplementary file 1 [file plants-12-02318-s001.zip › Figure S9 RNA polymerase.png]
